# A prognostic model for highly aggressive prostate cancer using interpretable machine learning techniques

**DOI:** 10.3389/fmed.2025.1512870

**Published:** 2025-05-12

**Authors:** Cong Peng, Cheng Gong, Xiaoya Zhang, Duxian Liu

**Affiliations:** Department of Pathology, The Second Hospital of Nanjing, Affiliated to Nanjing University of Chinese Medicine, Nanjing, Jiangsu, China

**Keywords:** prostate cancer, survival, machine, predictive analytics, Boruta algorithm

## Abstract

**Background:**

Extremely aggressive prostate cancer, including subtypes like small cell carcinoma and neuroendocrine carcinoma, is associated with poor prognosis and limited treatment options. This study sought to create a robust, interpretable machine learning-based model that predicts 1-, 3-, and 5-year survival in patients with extremely aggressive prostate cancer. Additionally, we sought to pinpoint key prognostic factors and their clinical implications through an innovative method.

**Materials and methods:**

This study retrospectively analyzed data from 1,620 patients with extremely aggressive prostate cancer in the SEER database (2000–2020). Feature selection was performed using the Boruta algorithm, and survival predictions were made using nine machine learning algorithms, including XGBoost, logistic regression (LR), support vector machine (SVM), random forest (RF), k-nearest neighbor (KNN), decision tree (DT), elastic network (Enet), multilayer perceptron (MLP) and lightGBM. Model performance was evaluated using metrics such as AUC, accuracy (F1 score), confusion matrix, and decision curve analysis. Additionally, Shapley Additive Explanations (SHAP) were applied to interpret feature importance within the model, revealing the clinical factors that influence survival predictions.

**Results:**

Among the nine models, the lightGBM model exhibited the best performance, with an AUC and F1 score of (0.8, 0.809) for 1-year survival prediction, (0.809, 0.751) for 3-year survival prediction, and (0.773, 0.611) for 5-year survival prediction. SHAP analysis revealed that M stage was the most important feature for predicting 1- and 3-year survival, while PSA level had the greatest impact on 5-year survival predictions. The model demonstrated good clinical utility and predictive accuracy through decision curve analysis and confusion matrix.

**Conclusion:**

The lightGBM model has good predictive power for survival in patients with extremely aggressive prostate cancer. By identifying key clinical factors and providing actionable predictions, the model has the potential to enhance prognostic accuracy and improve patient outcomes.

## Introduction

According to the Cancer Statistics 2024 published by the American Cancer Society, the United States is expected to diagnose approximately 299,010 new cases of prostate cancer in 2024, accounting for 14.9 percent of all new cancer cases. In addition, about 35,250 men are expected to die from prostate cancer in 2024, making it the second leading cause of cancer deaths among men in the United States (1). Extremely aggressive prostate adenocarcinoma, a rare subtype of prostate cancer, represents 5 to 10% of all prostate cancer cases (2). This category includes subtypes such as small cell carcinoma, squamous cell carcinoma, and neuroendocrine carcinoma, which are associated with higher metastatic rates and a worse prognosis (3, 4). In contrast to typical prostate adenocarcinomas, these aggressive forms are often resistant to standard hormonal therapies and present with widespread metastases at the time of diagnosis, leading to significantly reduced survival times (5, 6). Once metastasis occurs, the median survival for these patients is typically reported to be less than one year, and current treatment options show limited effectiveness (7, 8).

In recent years, machine learning, a burgeoning tool within the realm of artificial intelligence, has found extensive application in the medical field (9–11). By leveraging large-scale clinical datasets, machine learning can automatically detect and learn complex patterns, thereby enhancing the accuracy of disease prognosis predictions (9, 12). The latest review highlights how machine learning models are redefining the diagnosis and management of prostate cancer (13, 14).

Several previous studies have focused on developing machine-learning-based risk prediction models for prostate cancer. For example, Changhee et al. used machine learning to predict cancer-specific mortality in patients with non-metastatic prostate cancer. While Peng et al. developed a machine-learning-based prognostic model for patients with lymph node-positive prostate cancer. However, there is a lack of clinical tools for prognostic assessment of extremely aggressive prostate cancer patients with poor prognosis. Although traditional statistical models can provide some prognostic prediction, their ability to mine high-dimensional nonlinear data is limited and cannot fully reveal the relationship between complex biological features and prognostic outcomes (15, 16). Therefore, a novel predictive tool is needed to improve model performance and provide guidance for individualized treatment decisions. The innovation of this study is to combine Shap (Shapley Additive Explanations) with traditional machine learning, which breaks through the limitation of “black-boxing” of traditional machine learning models, and provides the importance scores of clinical variables for each prediction. This enables the model to not only provide highly accurate predictions but also quantify the specific impact of clinical variables on patient prognosis. This feature significantly improves the clinical usability of the model, and our study provides innovative ideas for the prognostic management of patients with extremely aggressive subtypes of prostate cancer.

## Methods

### Data source and patient selection

Patient information on extremely aggressive prostate cancer was obtained from the Surveillance, Epidemiology, and End Results (SEER) database, which covers approximately 30% of the U.S. population and is publicly accessible. We selected patients diagnosed between 2000 and 2020 with prostate cancer (ICD-O-3 code C61.9) who had pathological subtypes such as small cell carcinoma, large cell carcinoma, neuroendocrine carcinoma, squamous cell carcinoma, and aggressive ductal adenocarcinoma. Data extraction was performed using SEER*Stat software.

**Figure 1:**
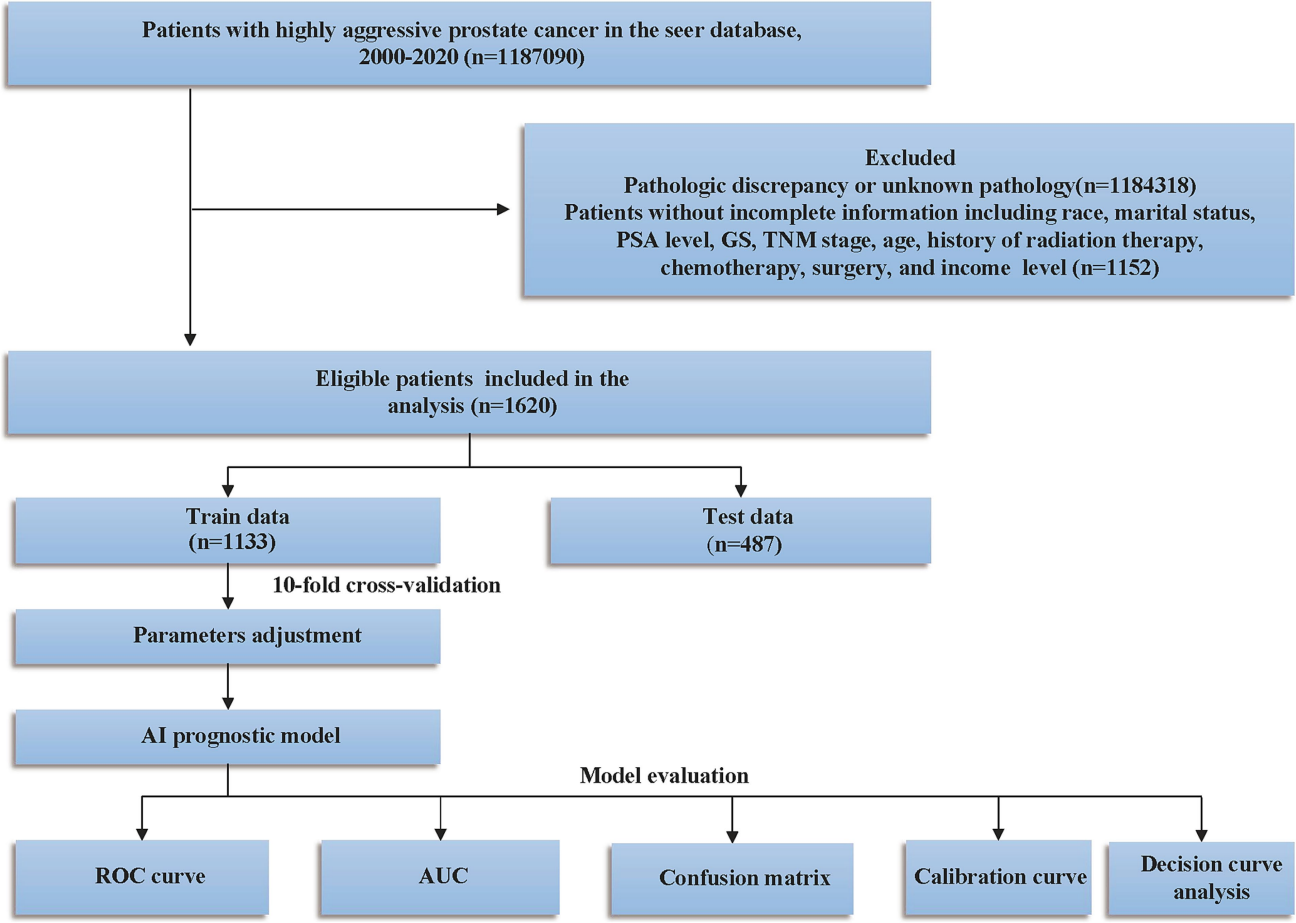
Study design and patient selection flowchart. PSA, prostate-specific antigen; GS, Gleason score; SEER, Surveillance, Epidemiology, and End Results; TNM, tumor lymph node metastasis; ROC, curve receiver operating characteristic curve; AUC, area under the curve.

The exclusion criteria were as follows: (1) mismatched pathological type; (2) patients with multiple primary tumors; and (3) patients with incomplete clinical information, such as missing data on race, survival, TNM stage, PSA level, Gleason score, or other key clinical variables. The inclusion and exclusion process are depicted in Figure 1.

### Study variables and feature selection

Data pertaining to demographics and clinical characteristics of prostate cancer patients were meticulously extracted from the SEER database. This encompassed variables such as age at diagnosis, race, gender, TNM stage as per the American Joint Committee on Cancer (AJCC) 7th edition, marital status, prostate-specific antigen (PSA) levels, Gleason score (GS), median household income, and various treatment modalities including surgery, radiotherapy, and chemotherapy. Following the categorization in previous studies (17, 18), age was divided into three groups: ≤60, 61–69, and ≥70 years. PSA levels were recorded as continuous variables, with values ≤0.1 ng/mL recorded as 0.1 ng/mL and values ≥98 ng/mL capped at 98 ng/mL, ranging from 0.1 to 98 ng/mL. Gleason scores were grouped into categories of ≤3 + 4, 4 + 3, 8, and ≥9. Missing data were addressed using the following strategies: for variables with missing rates below 20%, Random Forest Imputation was employed to estimate and fill in the missing values (19). Variables with more than 20% missing data were excluded from the analysis. In this study, all variables included in the analysis had missing rates below 20%. Among the variables included in the analysis, missing rates were as follows: Chemotherapy (4.2%), Marital status (6.8%), Income (3.1%), T stage (8.7%), N stage (7.3%) and M stage (4.1%). Random Forest Imputation (using the missForest package in R) was applied to ensure data completeness and consistency. For feature selection, we utilized the Boruta algorithm (20), which is a robust method for identifying the most significant features within a dataset. It determines feature importance by comparing the Z-scores of each actual feature against those of corresponding “shadow features.” In this process, all genuine features are duplicated and shuffled to create shadow features, which are then evaluated using a Random Forest model to obtain their respective Z-scores. Additionally, the Z-scores of the shadow features are generated by randomly permuting the original features (21). A true feature is deemed “important” (indicated in green) and classified as an acceptable variable if its Z-score consistently surpasses the maximum Z-score of the shadow features across multiple independent tests. Conversely, if a true feature’s Z-score does not significantly exceed that of the shadow features, it is labeled as “unimportant” (indicated in red) and classified as an unacceptable variable. Acceptable variables are retained during the feature selection process as they are considered to contribute positively to the model’s performance. In contrast, unacceptable variables are excluded from the final feature set because they do not demonstrate sufficient predictive capability for the target variable during the feature selection process.

### Model development

Prognostic models were constructed using nine machine learning algorithms: XGBoost, logistic regression (LR), support vector machine (SVM), random forest (RF), k-nearest neighbor (KNN), decision tree (DT), elastic network (Enet), multilayer perceptron (MLP), and lightGBM. To ensure model stability, the dataset was split into a 70:30 ratio for training and testing. Cross-validation was performed with 10-fold testing, and hyperparameters were tuned in the training set. Final validation was conducted on the test set. The objective was to develop models that could predict the overall survival of patients with extremely aggressive prostate cancer at 1, 3, and 5 years.

### Statistical analysis

Categorical variables were analyzed using the χ^2^ test and expressed as numbers (*n*) and percentages (%). Non-normally distributed continuous variables were assessed with the Kruskal-Wallis test and reported as medians with interquartile ranges (IQR). All statistical analyses and model development were conducted using R (version 4.0.5). A *p*-value of <0.05 was considered statistically significant.

### Model performance evaluation

The performance of the nine machine learning models was evaluated using receiver operating characteristic (ROC) curve analysis and confusion matrices. The area under the curve (AUC) of the ROC curve measures the performance of the model, and F1 scores combining sensitivity and specificity are used to assess the robustness of the model (22). Additionally, calibration curves based on Bier scores and decision curve analysis (DCA) were applied to assess the models’ prediction accuracy and clinical utility.

### Model interpretation

SHAP (Shapley Additive Explanations) values were used to interpret the machine learning models. SHAP values, derived from game theory, provide insights into which features most significantly influence the model’s predictions and how each feature affects the model’s output.

## Results

### Patient characteristics

1,620 patients were included in this study, and the baseline characteristics of the training set and test set are shown in Table 1. There was no difference between the training set and validation set in the baseline data. There were 1,133 columns of patients assigned to the training set and 487 columns of patients assigned to the validation set. In the training set 631 patients died and 502 patients survived. In the validation set 277 patients died and 210 patients survived.

**Table 1:** Baseline characteristics of extremely aggressive prostate cancer patients.

Characteristics	Training cohort (*n* = 1,133)	Validation cohort (*n* = 487)	*P* value
Age, yr. *n* (%)			0.53
≤60	197 (17.39)	96 (19.71)	
61–69	363 (32.04)	153 (31.42)	
≥70	573 (50.57)	238 (48.87)	
Race, *n* (%)			0.09
White	915 (80.76)	386 (79.26)	
Black	114 (10.06)	65 (13.35)	
Other^a^	104 (9.18)	36 (7.39)	
Clinical T stage, *n* (%)			0.36
T1	426 (37.6)	211 (43.33)	
T2	312 (27.54)	125 (25.67)	
T3	204 (18.01)	82 (16.84)	
T4	191 (16.86)	69 (14.17)	
*N*, *n* (%)			0.86
N0	931 (82.17)	398 (81.72)	
N1	202 (17.83)	89 (18.28)	
M, *n* (%)			0.46
M0	761 (67.17)	337 (69.20)	
M1	372 (32.83)	150 (30.80)	
Surgery, *n* (%)			0.69
No/Unknown	549 (48.46)	230 (47.23)	
Yes	584 (51.54)	257 (52.77)	
Radiation, *n* (%)			0.56
Yes	353 (31.16)	144 (29.57)	
No/Unknown	780 (68.84)	343 (70.43)	
Chemotherapy, *n* (%)			0.70
Yes	284 (25.07)	117 (24.02)	
No/Unknown	849 (74.93)	370 (75.98)	
Survival status, *n* (%)			0.69
Dead	631 (55.69)	277 (56.88)	
Alive	502 (44.31)	210 (43.12)	
Marital status, *n* (%)			0.66
Married	791 (69.81)	334 (68.58)	
Unmarried^b^	342 (30.19)	153 (31.42)	
Income, *n* (%)			0.35
≤100,000	947 (83.58)	397 (81.52)	
>100,000	186 (16.42)	90 (18.48)	
PSA level (ng/ml)			0.86
Median [IQR]	8.900 [4.700, 19.582]	9.000 [4.300, 20.499]	

PSA, prostate specific antigen; IQR, interquartile range; Other^a^: Asian/Pacific Islander, American Indian/Alaska Native. Unmarried^b^: Widowed, Divorced, Separated, Single (never married).

### Feature predictor selection

**Figure 2:**
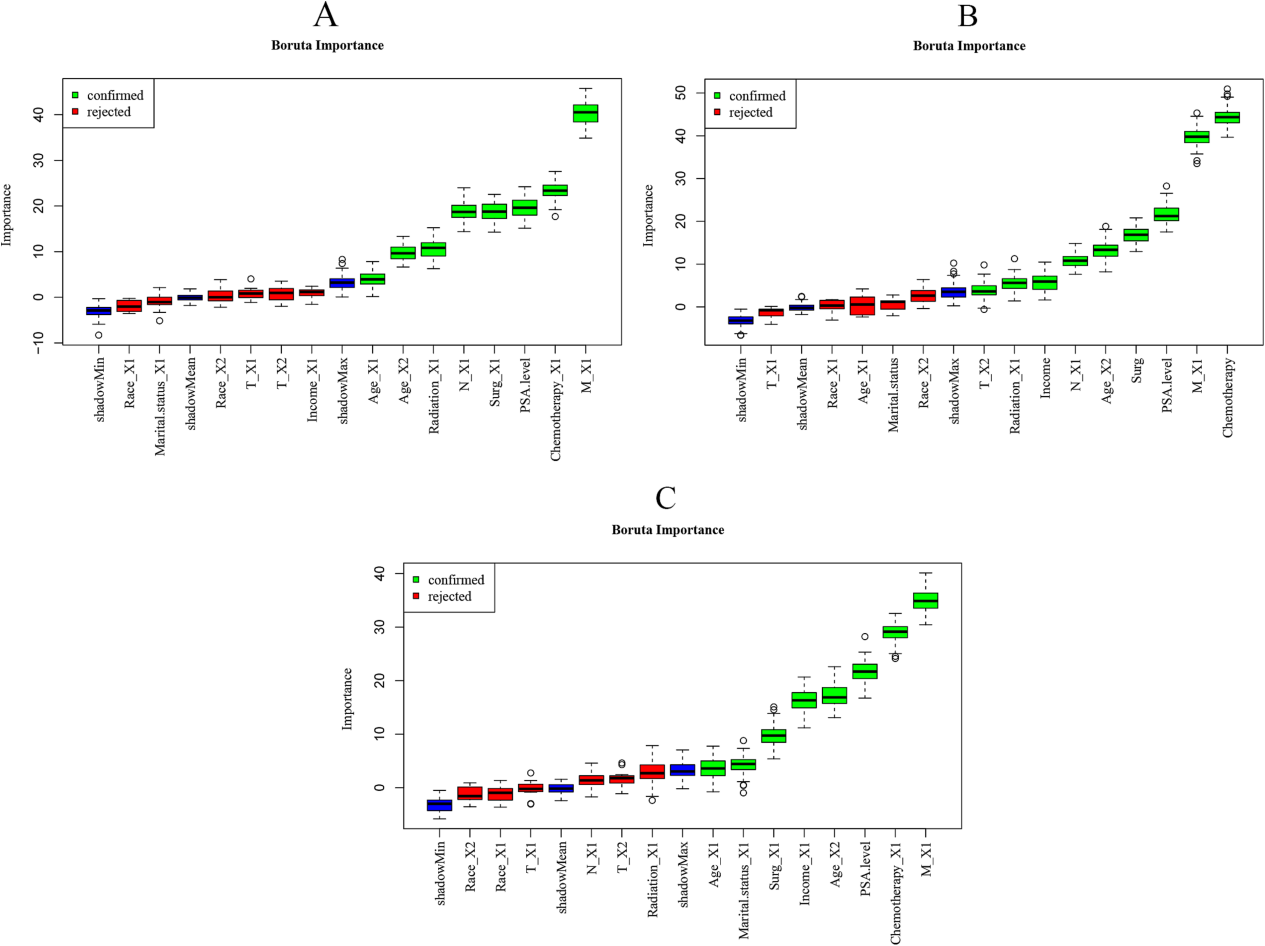
Importance of each feature in the predictive model based on Boruta’s algorithm. **(A)** Importance of each feature in the 1-year prognostic model. **(B)** Importance of each feature in the 3-year prognostic model. **(C)** Importance of each feature in the 5-year prognostic model. The Boruta algorithm determines the importance of a feature by comparing the Z-score of each actual feature with the corresponding “shadow feature.” A real feature is considered “important” (shown in green), whereas, if the Z-score of a real feature does not significantly exceed the Z-score of the shadow feature, it is marked as “not important” (shown in red) and classified as an unacceptable variable.

We use the same feature sets for our 1-, 3-, and 5-year prediction models. The Boruta algorithm identified unique feature sets for the 1-, 3-, and 5-year prediction models (Figure 2). The results showed that the feature variables included in the 1-year prognostic model were age, radiotherapy, N stage, surgery, PSA level, chemotherapy, and M stage (Figure 2A). Characteristic variables included in the 3-year prognostic model were T stage, radiotherapy, income level, N stage, age, PSA level, M stage and chemotherapy (Figure 2B). Characteristic variables included in the 5-year prognostic model were age, survival status, surgery, income, PSA level, chemotherapy, and M stage (Figure 2C).

### Construction of machine learning predictive models

Considering survival months as the prognostic state, we integrate the features selected by the appeal-based Boruta algorithm into the variable training model. In the training set species, we used 10-fold cross-validation for iteration and optimization and finally determined that the lightGBM model performs best. We adjusted the parameter balance to avoid data overfitting and finally identified the key hyperparameters. The key parameters of lightGBM are as follows: tree_depth = 1, trees = 458, learn_rate = 0.0059, mtry = 5, min_*n* = 10, loss_reduction = 0.291. See Supplementary material 1 for hyperparameters of the nine machine learning models.

### Evaluating machine learning prognostic models

**Figure 3:**
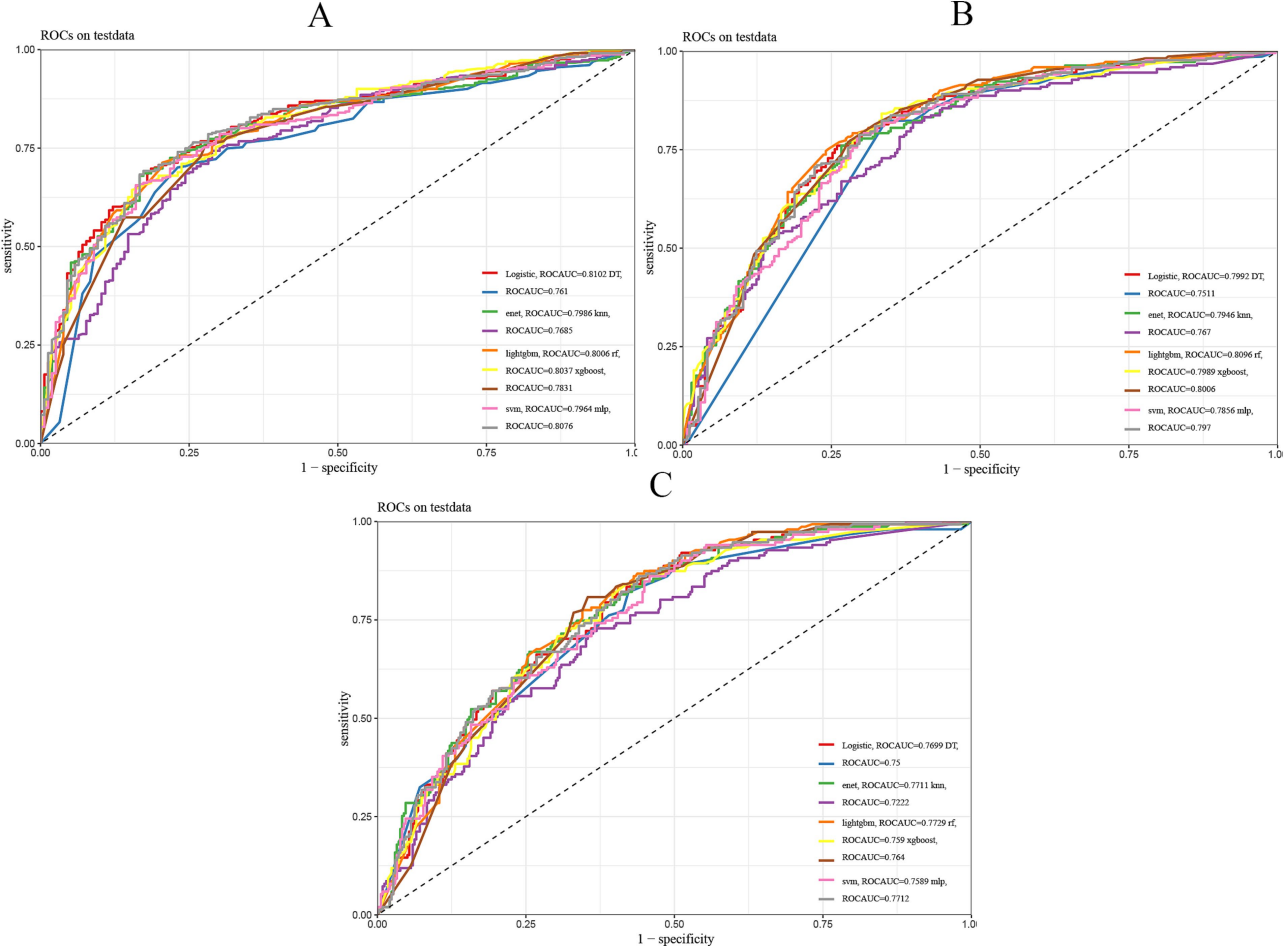
Nine machine learning models evaluated. **(A)** ROC curves of 1-year prognostic models in the test set. **(B)** ROC curves of 3-year prognostic models in the test set. **(C)** ROC curves of 5-year prognostic models in the test set. The plot presents the ROC curves for nine different machine learning models in a prediction task. The x-axis represents the false positive rate (FPR), and the y-axis represents the true positive rate (TPR). The area under the curve (AUC) reflects the overall performance of each model, with a larger AUC indicating better predictive ability.

Our analysis revealed that lightGBM demonstrated consistent efficacy in forecasting highly aggressive prostate cancer at 1, 3, and 5 years, as evidenced by the AUC values derived from the ROC curves of both the training and test sets. Data for 1 year (0.777 for the training set, 0.8 for the test set), 3 years (0.881 for the training set, 0.809 for the test set), and 5 years (0.888 for the training set, 0.773 for the test set) are presented in Figure 3 and Table 2.

**Table 2:** Performance of predictive models built by 9 machine learning algorithms in training and test sets (area under the ROC curve).

	1-year survival	3-year survival	5-year survival
Train set			
LightGBM	0.777	0.881	0.888
DT	0.856	0.782	0.853
ENET	0.768	0.782	0.853
KNN	0.909	0.788	0.805
Logistic	0.776	0.805	0.824
MLP	0.777	0.869	0.862
RF	0.852	0.796	0.819
SVM	0.779	0.802	0.807
XGBoost	0.763	0.799	0.808
Test set			
LightGBM	0.800	0.809	0.773
DT	0.761	0.751	0.75
ENET	0.798	0.795	0.771
KNN	0.769	0.767	0.722
Logistic	0.810	0.799	0.769
MLP	0.808	0.797	0.771
RF	0.804	0.798	0.759
SVM	0.796	0.786	0.758
XGBoost	0.783	0.800	0.764

DT, decision tree; ENET, Elastic Net; KNN, K-Nearest Neighbors; LightGBM, Light Gradient Boosting Machine; RF, Random Forest; XGBoost, Extreme Gradient Boosting; SVM, Support Vector Machine; MLP, Multi-Layer Perceptron.

See Table 2 for the best and most stable performance of lightGBM compared to the other 8 machine learning models. In addition, we evaluated the accuracy of the lightGBM model using a confusion matrix (Supplementary Figure 1). For 1-year, 3-year and 5-year survival predictions, F1 scores of lightGBM model validation set are 0.809, 0.751 and 0.611, respectively (Supplementary Table 1). Therefore, lightGBM model has the best predictive performance in 3-year and 5-year models. Although the one-year survival prediction is slightly lower than that of Logistic, MLP and RF models, the stability of LightGBM model is superior to these three models. In summary, we choose LightGBM model as the best model.

**Figure 4:**
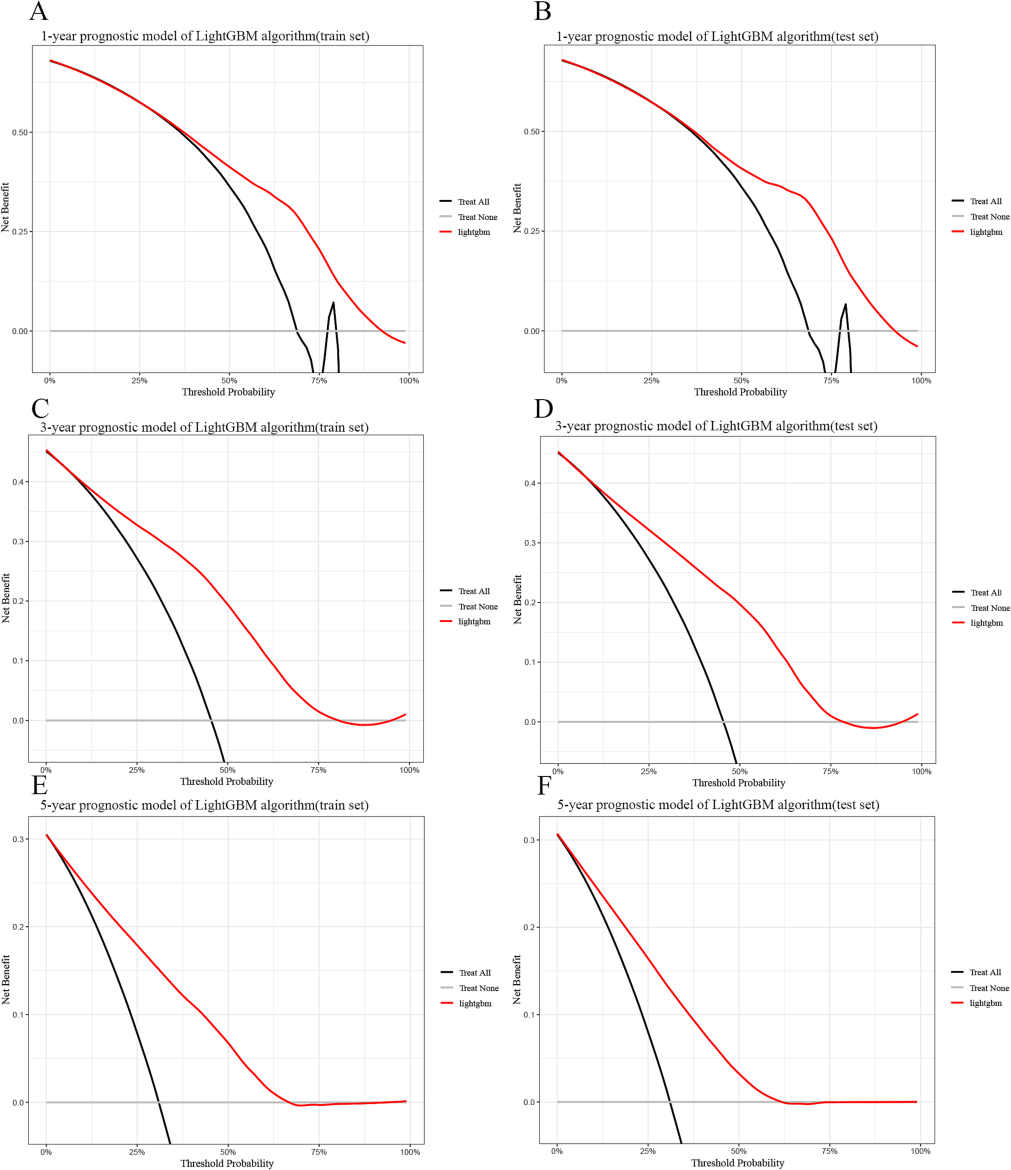
Decision curve analysis curves for the LightGBM model for the training and test sets. **(A)** 1-year train set. **(B)** 1-year test set. **(C)** 3-year train set. **(D)** 3-year test set. **(E)** 5-year train set. **(F)** 5-year test set. LightGBM: Light Gradient Boosting Machine. In the figure, the red curve represents the predicted performance of the GBM model, respectively. In addition, there are two lines, which represent two extreme cases. The gray vertical line indicates the assumption of survival for all patients. The black horizontal line indicates that there is no survival assumption. For example, in the 1-year training set, the survival probability is between 0.3 and 0.93. When using this GBM predictive model to make clinical decisions, survival probabilities can be distinguished.

Finally, we used calibration curves based on Bier scores showing that the predictions of 1-, 3-, and 5-year survival probabilities in the train and test sets were also more consistent with the actual observations (Supplementary Figures 2, 3). Also, DCA decision curve analysis showed good clinical utility and positive net benefit of lightGBM in 1, 3, 5-year survival prediction (Figure 4).

### Interpretation of models

**Figure 5:**
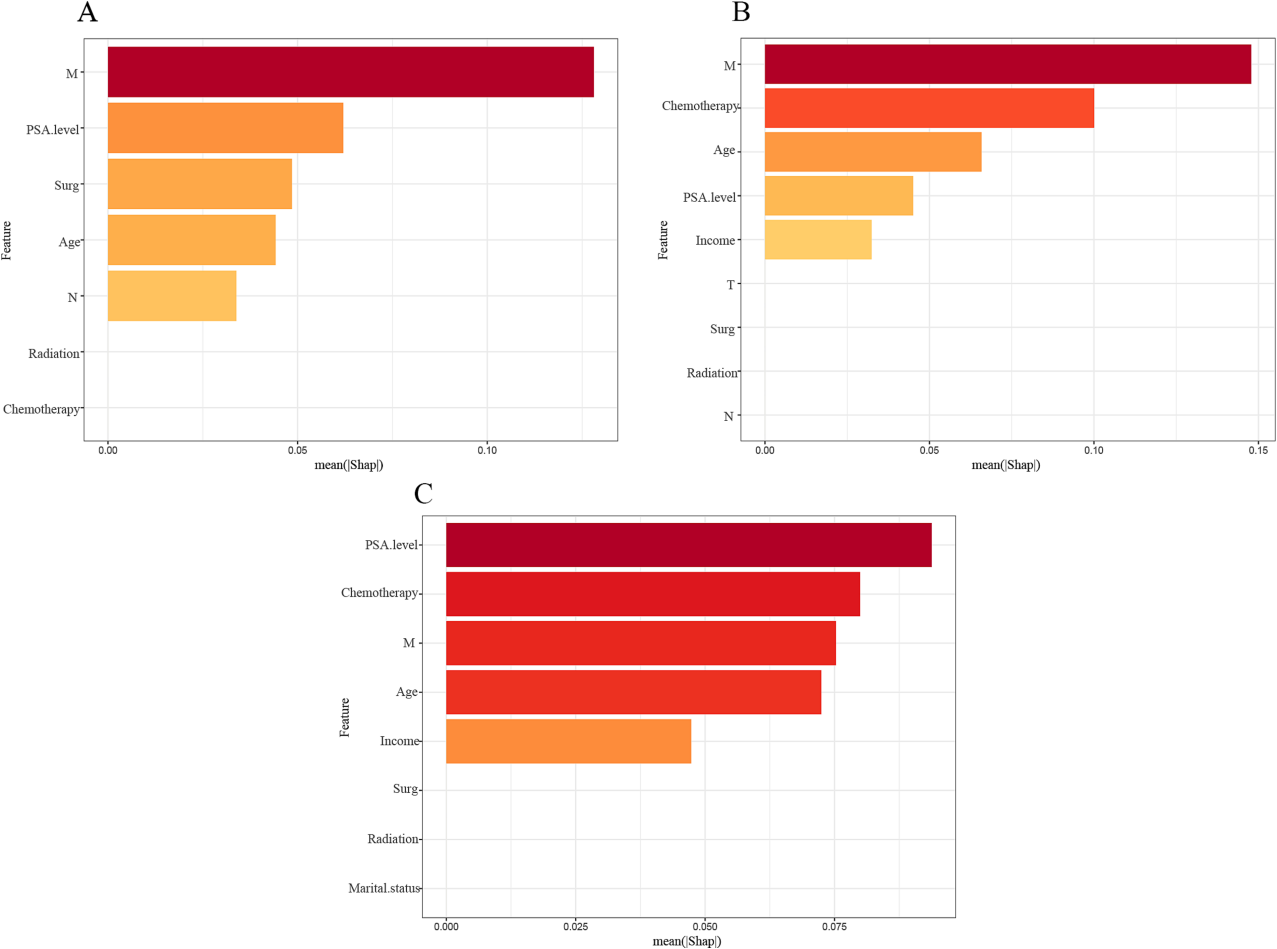
Importance ranking of features based on SHAP values in diagnostic models based on LightGBM algorithm. The features are ranked based on the sum of SHAP values of all the patients, and the distribution of the impact of each feature on the output of the model LightGBM is expressed in terms of the SHAP value. The x-axis represents the SHAP value’s impact on the model’s output. The higher the value of the x-axis, the greater the impact on the model. **(A)** 1-year model; **(B)** 3-year model; **(C)** 5-year model.

These key features were ranked using a SHAP plot (Figure 5) showing the level of influence of the machine learning model for each feature. The SHAP plot showed that the largest factor influencing patient survival at 1 and 3 years was M stage and the largest factor influencing patient survival at 5 years was PSA level.

### Application of model

To facilitate clinical adoption, we have uploaded the R code, dataset, and the completed model to Supplementary material 3. Additionally, we propose integrating this model into hospital electronic health records (EHRs) and clinical decision support systems (CDSS) to assist oncologists in real-time prognostic estimation.

## Discussion

Patients with extremely aggressive prostate cancer, including small cell carcinoma, large cell carcinoma, squamous cell carcinoma, neuroendocrine carcinoma, undifferentiated carcinoma, aggressive ductal carcinoma, and ductal adenocarcinoma, often exhibit more aggressive biological behavior and have a poorer prognosis compared to other forms of prostate cancer (23–25). Accurate survival prediction for these patients is therefore clinically significant. However, current clinical tools for prognostic prediction in extremely aggressive prostate cancer have substantial limitations, particularly the absence of reliable models that leverage artificial intelligence and machine learning.

This research involved the creation of nine models grounded in machine learning to forecast survival rates at 1, 3, and 5 years for the patient cohort in question. Among these, the lightGBM model showed the highest predictive performance, with AUCs of 0.77, 0.80, 0.88, and 0.81 for the training and test sets at 1, 3, and 5 years, respectively, demonstrating strong predictive ability. An AUC value of ≥0.7 is considered indicative of a model with sufficient predictive power (26).

In recent years, artificial intelligence has garnered increasing attention in the medical field, including in prostate cancer research (27–30). In contrast to conventional algorithms, machine learning models operate without the limitations imposed by non-proportionality, multicollinearity, or nonlinearity challenges (30). Thereby minimizing biases that can arise from conventional modeling. For example, Peng et al. used machine learning algorithms to develop a survival prognostic model for patients with lymph node-positive prostate cancer, achieving better predictive performance than traditional Cox regression models (31). Similarly, Dai et al. (32) demonstrated that machine learning models outperformed traditional algorithms in predicting survival for patients with confined prostate cancer.

In this study, we incorporated 12 key clinical characteristics of patients with extremely aggressive prostate cancer and used the Boruta algorithm, a feature selection method based on random forest classifiers, to select the most relevant features for survival prediction. The Boruta algorithm is designed to identify all variables that are important to the dependent variable, rather than the smallest set of features relevant to a particular model (33, 34). In contrast to the objective of a typical feature selection algorithm, the Boruta feature selection algorithm aims to identify the features that hold the greatest relevance to the dependent variable, rather than merely seeking the most compact set of features pertinent to a specific model (34). Our results identified factors such as age, PSA level, surgery, and radiotherapy as key risk factors for prognosis, with tumor metastasis (M stage) emerging as the most significant predictor of survival at 1 and 3 years, and PSA level as the strongest predictor at 5 years. These findings have important clinical implications. For example, the model highlights surgery and radiotherapy as influential factors, suggesting that multimodal treatment approaches may provide survival benefits in certain subgroups of patients with highly aggressive prostate cancer. This underscores the need for personalized treatment selection based on a patient’s predicted prognosis and treatment response patterns.

A systematic review identified high Gleason scores as independent risk factors for early tumor progression, and multiple organ metastases were associated with reduced survival (35). In a separate investigation, the median overall survival for patients newly diagnosed with neuroendocrine prostate cancer was recorded at 16.8 months, significantly less than the 53.5 months noted in cases associated with treatment (36). Regarding treatment, platinum-based chemotherapy is commonly used for patients with small cell carcinoma. Combination regimens including cisplatin, etoposide, and doxorubicin have shown partial benefit, though they are not recommended for neuroendocrine prostate cancer patients due to the risk of severe neutropenia. For neuroendocrine prostate cancer, immune checkpoint inhibitors, such as atezolizumab combined with platinum-based chemotherapy (36) or second-line treatments such as natalizumab with ibritumomab may be considered (37).

Early detection of prostate cancer is critical. Various non-invasive imaging techniques have been studied for predicting metastasis (38–40). Multiparametric MRI (mpMRI) has shown enhanced sensitivity and specificity relative to conventional MRI in the identification of tumors and lymph nodes; however, it may experience signal loss or image distortion in DWI sequences (39). Similarly, PSMA PET/CT is extensively utilized for the detection of prostate cancer in both soft tissue and bone, yet its detection rate for lymph node metastases measuring 2–5 mm hovers around 60% (40, 41). Emerging imaging techniques, such as MR lymphography and targeted PET using superparamagnetic iron oxide (SPIO) nanoparticles, are under investigation, though their effectiveness in predicting lymph node metastasis remains uncertain (41–43). Furthermore, fluid-based diagnostics, exemplified by the FDA-approved Prostate Cancer Antigen 3 (PCA3), which is a urine-based, non-coding RNA biomarker, have demonstrated promise in informing decisions regarding repeat biopsies, with reported AUCs varying from 0.64 to 0.762 (43, 44). Other urine-based genomic assays, including multigene panels (e.g., PUR), exosome-based assays (e.g., ExoDx), DNA methylation markers (e.g., epiCaPture), and mRNA-based assays (e.g., SelectMDx), have also demonstrated prognostic value (44, 45). Lih et al. (46) identified urinary glycopeptides, such as ACPP, CLU, ORM1, and CD97, that may help differentiate between low- and high-risk prostate cancer, showing potential for early identification of aggressive forms of the disease.

This study is the first to develop multiple machines learning prognostic models specifically for extremely aggressive prostate cancer. We incorporated 13 significant prognostic features and employed SHAP values to assess the contribution of each feature, revealing that metastasis, surgery, and PSA level were the most impactful variables.

However, this study has several limitations that should be acknowledged. First, as a retrospective study utilizing SEER data, it may be subject to selection bias and incomplete case reporting, potentially affecting the generalizability of our findings. Second, the SEER database does not provide detailed molecular markers, genetic data, or treatment response information, which are critical for a more comprehensive prognostic assessment. The absence of these key clinical variables may limit the ability of our model to fully capture the biological heterogeneity of extremely aggressive prostate cancer. Future studies should aim to incorporate multi-omics data and real-world patient responses to further refine predictive accuracy. Additionally, while our model has demonstrated strong internal validation, external validation on independent datasets and prospective clinical trials are needed to ensure its applicability across diverse populations.

Overall, this study highlights the potential of machine learning models to guide clinical decisions and optimize treatment strategies for extremely aggressive prostate cancer. Specifically, our model can be used for risk stratification and treatment planning of patients, as well as monitoring and follow-up adjustments for patients at different risks, and finally, by integrating the model into EHRs and CDSS, can provide real-time survival predictions to help physicians make evidence-based treatment recommendations. With the accumulation of more clinical data and further optimization of algorithms, AI-based prognostic models could significantly improve treatment outcomes and survival for patients with extremely aggressive prostate cancer in the future.

## Conclusion

In conclusion, we developed and evaluated nine machine learning models, incorporating SHAP values to enhance interpretability, for predicting survival in patients with extremely aggressive prostate cancer. Among them, the lightGBM model demonstrated the best predictive performance, offering a valuable clinical tool for personalized prognosis estimation. Future research should focus on external validation using independent cohorts, integrating molecular biomarkers, and exploring the incorporation of real-time patient data to further enhance the model’s robustness and clinical utility.

## Data Availability

Publicly available datasets were analyzed in this study. This data can be found at: https://seer.cancer.gov/.
