# Supplementary material for: A prognostic model for highly aggressive prostate cancer using interpretable machine learning techniques
Source: Front Med (Lausanne). 2025 May 12;12:1512870. doi: 10.3389/fmed.2025.1512870 (PMC12104253; doi:10.3389/fmed.2025.1512870)
Supplement: Supplementary file 1 [file Data_Sheet_1.PDF]

Supplementary R Script for Data Processing and Model Comparison

Your Name *

December 7, 2024

1 Introduction

This supplementary document contains the R script used for comparing multiple machine learning algorithms using parallel computation. The script includes libraries for parallel computing, data preprocessing, and model evaluation.

2 R Script

The following R script demonstrates how to load libraries, set up parallel computation, read data, preprocess it, and apply various machine learning algorithms:

```
#1.
library(tidymodels)
library(bonsai)
setwd("D:/R work")
library(doParallel)
cl <- makePSOCKcluster(12)
registerDoParallel(cl)




#2.
rawdata <- readr::read_csv(file.choose())
colnames(rawdata)
for(i in c(2:9)){ rawdata[[i]] <- factor(rawdata[[i]])}
skimr::skim(rawdata)



#3.
yourpositivelevel <- "Yes"
yournegativelevel <- "No"
```

*Your Institution, Your Department, Your Country. Email: your.email@example.com.

```
levels(rawdata$Survival.months)
table(rawdata$Survival.months)
rawdata$Survival.months <- factor(
  rawdata$Survival.months,
  levels = c(yournegativelevel, yourpositivelevel)
)
levels(rawdata$Survival.months)
table(rawdata$Survival.months)



#4.
set.seed(2023)
datasplit <- initial_split(rawdata, prop = 0.7, strata =
   Survival.months)
traindata <- training(datasplit)
testdata <- testing(datasplit)



#5.
set.seed(2023)
folds <- vfold_cv(traindata, v =10, strata = Survival.months)
folds



new_dummy_names <- function (var, lvl, ordinal = FALSE) {
  args <- vctrs::vec_recycle_common(var, lvl)
  var <- args[[1]]
  lvl <- args[[2]]
  nms <- paste(var, lvl, sep = "_")
  nms
}



#6.

datarecipe_dt <- recipe(formula = Survival.months ~ PSA.level
    + Age + M + Radiation + Chemotherapy + Marital.status +
   Income + Surg, traindata)


datarecipe_rf <- recipe(formula = Survival.months ~ PSA.level
    + Age + M + Radiation + Chemotherapy + Marital.status +
   Income + Surg, traindata)
```

```
datarecipe_xgboost <- recipe(Survival.months ~ PSA.level + Age + M + Radiation + Chemotherapy + Marital.status + Income + Surg, traindata) %>%
  step_dummy(all_nominal_predictors(), naming = new_dummy_names)

datarecipe_lightgbm <- recipe(Survival.months ~ PSA.level + Age + M + Radiation + Chemotherapy + Marital.status + Income + Surg, traindata) %>%
  step_dummy(all_nominal_predictors(), naming = new_dummy_names)


datarecipe_enet <- recipe(formula = Survival.months ~ PSA.level + Age + M + Radiation + Chemotherapy + Marital.status + Income + Surg, traindata) %>%
  step_dummy(all_nominal_predictors(), naming = new_dummy_names) %>%
  step_normalize(all_predictors())


datarecipe_svm <- recipe(formula = Survival.months ~ PSA.level + Age + M + Radiation + Chemotherapy + Marital.status + Income + Surg, traindata) %>%
  step_dummy(all_nominal_predictors(), naming = new_dummy_names) %>%
  step_normalize(all_predictors())


datarecipe_mlp <- recipe(formula = Survival.months ~ PSA.level + Age + M + Radiation + Chemotherapy + Marital.status + Income + Surg, traindata) %>%
  step_dummy(all_nominal_predictors(), naming = new_dummy_names) %>%
  step_range(all_predictors())


datarecipe_knn <- recipe(formula = Survival.months ~ PSA.level + Age + M + Radiation + Chemotherapy + Marital.status + Income + Surg, traindata) %>%
  step_dummy(all_nominal_predictors(), naming = new_dummy_names) %>%
  step_normalize(all_predictors())


datarecipe_logistic <- recipe(formula = Survival.months ~ PSA
```

```
    .level + Age + M + Radiation + Chemotherapy + Marital.
    status + Income + Surg, traindata)



#7.
model_dt <- decision_tree(
  mode = "classification",
  engine = "rpart",
  tree_depth = tune(),
  min_n = tune(),
  cost_complexity = tune()
) %>%
  set_args(model=TRUE)
model_dt


model_rf <- rand_forest(
  mode = "classification",
  engine = "randomForest", # ranger
  mtry = tune(),
  trees = tune(),
  min_n = tune()
) %>%
  set_args(importance = T)
model_rf



model_xgboost <- boost_tree(
  mode = "classification",
  engine = "xgboost",
  mtry = tune(),
  trees = 1000,
  min_n = tune(),
  tree_depth = tune(),
  learn_rate = tune(),
  loss_reduction = tune(),
  sample_size = tune(),
  stop_iter = 25
) %>%
  set_args(validation = 0.2,
           event_level = "second")
model_xgboost
```

```
model_lightgbm <- boost_tree(
  mode = "classification",
  engine = "lightgbm",
  tree_depth = tune(),
  trees = tune(),
  learn_rate = tune(),
  mtry = tune(),
  min_n = tune(),
  loss_reduction = tune()
)
model_lightgbm



model_enet <- logistic_reg(
  mode = "classification",
  engine = "glmnet",
  mixture = tune(),
  penalty = tune()
)
model_enet



model_mlp <- mlp(
  mode = "classification",
  engine = "nnet",
  hidden_units = tune(),
  penalty = tune(),
  epochs = tune()
)
model_mlp



model_svm <- svm_rbf(
  mode = "classification",
  engine = "kernlab",
  cost = tune(),
  rbf_sigma = tune()
)
model_svm
```

```
model_knn <- nearest_neighbor(
  mode = "classification",
  engine = "kknn",
  neighbors = tune(),
  weight_func = tune(),
  dist_power = 2
)
model_knn



model_logistic <- logistic_reg(
  mode = "classification",
  engine = "glm"
)
model_logistic






#8.
wk_dt <-
  workflow() %>%
  add_recipe(datarecipe_dt) %>%
  add_model(model_dt)
wk_dt


wk_rf <-
  workflow() %>%
  add_recipe(datarecipe_rf) %>%
  add_model(model_rf)
wk_rf


wk_xgboost <-
  workflow() %>%
  add_recipe(datarecipe_xgboost) %>%
  add_model(model_xgboost)
wk_xgboost

wk_lightgbm <-
  workflow() %>%
  add_recipe(datarecipe_lightgbm) %>%
  add_model(model_lightgbm)
```

```
wk_lightgbm

wk_enet <-
  workflow() %>%
  add_recipe(datarecipe_enet) %>%
  add_model(model_enet)
wk_enet

wk_mlp <-
  workflow() %>%
  add_recipe(datarecipe_mlp) %>%
  add_model(model_mlp)
wk_mlp


wk_svm <-
  workflow() %>%
  add_recipe(datarecipe_svm) %>%
  add_model(model_svm)
wk_svm

wk_knn <-
  workflow() %>%
  add_recipe(datarecipe_knn) %>%
  add_model(model_knn)
wk_knn

wk_logistic <-
  workflow() %>%
  add_recipe(datarecipe_logistic) %>%
  add_model(model_logistic)
wk_logistic







#9.
set.seed(2023)
hpgrid_dt <- parameters(
  tree_depth(range = c(3, 7)),
  min_n(range = c(5, 10)),
  cost_complexity(range = c(-6, -3))
) %>%
  grid_random(size = 20)
hpgrid_dt
```

```
log10(hpgrid_dt$cost_complexity)




set.seed(2023)
hpgrid_rf <- parameters(
  mtry(range = c(2, 10)),
  trees(range = c(200, 500)),
  min_n(range = c(20, 50))
) %>%
  grid_random(size = 20) #
hpgrid_rf





set.seed(2023)
hpgrid_xgboost <- parameters(
  mtry(range = c(2, 8)),
  min_n(range = c(5, 20)),
  tree_depth(range = c(1, 3)),
  learn_rate(range = c(0.01, 0.3)),
  loss_reduction(range = c(-3, 0)),
  sample_prop(range = c(0.8, 1))
) %>%
  grid_random(size = 20) #
hpgrid_xgboost



set.seed(2023)
hpgrid_lightgbm <- parameters(
  tree_depth(range = c(1, 3)),
  trees(range = c(100, 500)),
  learn_rate(range = c(-3, -1)),
  mtry(range = c(2, 8)),
  min_n(range = c(5, 10)),
  loss_reduction(range = c(-3, 0))
) %>%
  grid_random(size = 20) #
hpgrid_lightgbm




set.seed(2023)
```

```
hpgrid_enet <- parameters(
  mixture(),
  penalty(range = c(-5, 0))
) %>%
  grid_regular(levels = c(5, 20))
hpgrid_enet





set.seed(2023)
hpgrid_mlp <- parameters(
  hidden_units(range = c(15, 24)),
  penalty(range = c(-3, 0)),
  epochs(range = c(50, 150))
) %>%
  grid_regular(levels = 3) #
hpgrid_mlp







set.seed(2023)
hpgrid_svm <- parameters(
  cost(range = c(-5, 5)),
  rbf_sigma(range = c(-4, -1))
) %>%
  grid_random(size = 20) #
hpgrid_svm





set.seed(2023)
hpgrid_knn <- parameters(
  neighbors(range = c(3, 11)),
  weight_func()
) %>%
  grid_random(size = 20) #
hpgrid_knn
```

```
set.seed(2023)
tune_dt <- wk_dt %>%
  tune_grid(
    resamples = folds,
    grid = hpgrid_dt,
    metrics = metric_set(yardstick::accuracy,
                         yardstick::roc_auc,
                         yardstick::pr_auc),
    control = control_grid(save_pred = T,
                           verbose = T,
                           event_level = "second",
                           parallel_over = "everything",
                           save_workflow = T)
  )


set.seed(2023)
tune_rf <- wk_rf %>%
  tune_grid(
    resamples = folds,
    grid = hpgrid_rf,
    metrics = metric_set(yardstick::accuracy,
                         yardstick::roc_auc,
                         yardstick::pr_auc),
    control = control_grid(save_pred = T,
                           verbose = T,
                           event_level = "second",
                           parallel_over = "everything",
                           save_workflow = T)
  )


set.seed(2023)
tune_xgboost <- wk_xgboost %>%
  tune_grid(
    resamples = folds,
    grid = hpgrid_xgboost,
    metrics = metric_set(yardstick::accuracy,
                         yardstick::roc_auc,
                         yardstick::pr_auc),
    control = control_grid(save_pred = T,
                           verbose = T,
                           event_level = "second",
                           parallel_over = "everything",
```

```
                              save_workflow = T)
  )


set.seed(2023)
tune_lightgbm <- wk_lightgbm %>%
  tune_grid(
    resamples = folds,
    grid = hpgrid_lightgbm,
    metrics = metric_set(yardstick::accuracy,
                          yardstick::roc_auc,
                          yardstick::pr_auc),
    control = control_grid(save_pred = T,
                            verbose = T,
                            event_level = "second",
                            parallel_over = "everything",
                            save_workflow = T)
  )


set.seed(2023)
tune_enet <- wk_enet %>%
  tune_grid(
    resamples = folds,
    grid = hpgrid_enet,
    metrics = metric_set(yardstick::accuracy,
                          yardstick::roc_auc,
                          yardstick::pr_auc),
    control = control_grid(save_pred = T,
                            verbose = T,
                            event_level = "second",
                            parallel_over = "everything",
                            save_workflow = T)
  )

set.seed(2023)
tune_mlp <- wk_mlp %>%
  tune_grid(
    resamples = folds,
    grid = hpgrid_mlp,
    metrics = metric_set(yardstick::accuracy,
                          yardstick::roc_auc,
                          yardstick::pr_auc),
    control = control_grid(save_pred = T,
                            verbose = T,
                            event_level = "second",
                            parallel_over = "everything",
                            save_workflow = T)
```

```
  )


set.seed(2023)
tune_svm <- wk_svm %>%
  tune_grid(
    resamples = folds,
    grid = hpgrid_svm,
    metrics = metric_set(yardstick::accuracy,
                         yardstick::roc_auc,
                         yardstick::pr_auc),
    control = control_grid(save_pred = T,
                           verbose = T,
                           event_level = "second",
                           parallel_over = "everything",
                           save_workflow = T)
  )


set.seed(2023)
tune_knn <- wk_knn %>%
  tune_grid(
    resamples = folds,
    grid = hpgrid_knn,
    metrics = metric_set(yardstick::accuracy,
                         yardstick::roc_auc,
                         yardstick::pr_auc),
    control = control_grid(save_pred = T,
                           verbose = T,
                           event_level = "second",
                           parallel_over = "everything",
                           save_workflow = T)
  )


#11.

eval_tune_dt <- tune_dt %>%
  collect_metrics()
eval_tune_dt
hpbest_dt <- tune_dt %>%
  select_by_one_std_err(metric = "roc_auc", desc(cost_
     complexity))
hpbest_dt


eval_tune_rf <- tune_rf %>%
  collect_metrics()
```

```
eval_tune_rf
hpbest_rf <- tune_rf %>%
  select_by_one_std_err(metric = "roc_auc", desc(min_n))
hpbest_rf


eval_tune_xgboost <- tune_xgboost %>%
  collect_metrics()
eval_tune_xgboost
hpbest_xgboost <- tune_xgboost %>%
  select_by_one_std_err(metric = "roc_auc", desc(min_n))
hpbest_xgboost


eval_tune_lightgbm <- tune_lightgbm %>%
  collect_metrics()
eval_tune_lightgbm
hpbest_lightgbm <- tune_lightgbm %>%
  select_by_one_std_err(metric = "roc_auc", desc(min_n))
hpbest_lightgbm


eval_tune_enet <- tune_enet %>%
  collect_metrics()
eval_tune_enet
hpbest_enet <- tune_enet %>%
  select_by_one_std_err(metric = "roc_auc", desc(penalty))
hpbest_enet


eval_tune_mlp <- tune_mlp %>%
  collect_metrics()
eval_tune_mlp
hpbest_mlp <- tune_mlp %>%
  select_by_one_std_err(metric = "roc_auc", desc(penalty))
hpbest_mlp


eval_tune_svm <- tune_svm %>%
  collect_metrics()
eval_tune_svm
hpbest_svm <- tune_svm %>%
  select_best(metric = "roc_auc")
hpbest_svm


eval_tune_knn <- tune_knn %>%
  collect_metrics()
```

```
eval_tune_knn
hpbest_knn <- tune_knn %>%
  select_by_one_std_err(metric = "roc_auc", desc(neighbors))
hpbest_knn



#12.
#
```

```
set.seed(2023)
final_dt <- wk_dt %>%
  finalize_workflow(hpbest_dt) %>%
  fit(traindata)
final_dt


set.seed(2023)
final_rf <- wk_rf %>%
  finalize_workflow(hpbest_rf) %>%
  fit(traindata)
final_rf


set.seed(2023)
final_xgboost <- wk_xgboost %>%
  finalize_workflow(hpbest_xgboost) %>%
  fit(traindata)
final_xgboost


set.seed(2023)
final_lightgbm <- wk_lightgbm %>%
  finalize_workflow(hpbest_lightgbm) %>%
  fit(traindata)
final_lightgbm

set.seed(2023)
final_enet <- wk_enet %>%
  finalize_workflow(hpbest_enet) %>%
  fit(traindata)
final_enet

set.seed(2023)
final_mlp <- wk_mlp %>%
  finalize_workflow(hpbest_mlp) %>%
  fit(traindata)
```

```
final_mlp

set.seed(2023)
final_svm <- wk_svm %>%
  finalize_workflow(hpbest_svm) %>%
  fit(traindata)
final_svm

set.seed(2023)
final_knn <- wk_knn %>%
  finalize_workflow(hpbest_knn) %>%
  fit(traindata)
final_knn

final_logistic <- wk_logistic %>%
  fit(traindata)
final_logistic





#13

evalcv_dt <- bestcv4cls2(
  wkflow = wk_dt,
  tuneresult = tune_dt,
  hpbest = hpbest_dt,
  yname = "Survival.months",
  modelname = "DT",
  v = 10,
  positivelevel = yourpositivelevel
)
evalcv_dt$cvroc
evalcv_dt$cvpr
evalcv_dt$evalcv


evalcv_rf <- bestcv4cls2(
  wkflow = wk_rf,
  tuneresult = tune_rf,
  hpbest = hpbest_rf,
  yname = "Survival.months",
  modelname = "rf",
  v = 10,
  positivelevel = yourpositivelevel
)
evalcv_rf$cvroc
```

```
evalcv_rf$cvpr
evalcv_rf$evalcv


evalcv_xgboost <- bestcv4cls2(
  wkflow = wk_xgboost,
  tuneresult = tune_xgboost,
  hpbest = hpbest_xgboost,
  yname = "Survival.months",
  modelname = "xgboost",
  v = 10,
  positivelevel = yourpositivelevel
)
evalcv_xgboost$cvroc
evalcv_xgboost$cvpr
evalcv_xgboost$evalcv


evalcv_lightgbm <- bestcv4cls2(
  wkflow = wk_lightgbm,
  tuneresult = tune_lightgbm,
  hpbest = hpbest_lightgbm,
  yname = "Survival.months",
  modelname = "lightgbm",
  v = 10,
  positivelevel = yourpositivelevel
)
evalcv_lightgbm$cvroc
evalcv_lightgbm$cvpr
evalcv_lightgbm$evalcv



evalcv_enet <- bestcv4cls2(
  wkflow = wk_enet,
  tuneresult = tune_enet,
  hpbest = hpbest_enet,
  yname = "Survival.months",
  modelname = "enet",
  v = 10,
  positivelevel = yourpositivelevel
)
evalcv_enet$cvroc
evalcv_enet$cvpr
evalcv_enet$evalcv
```

```
evalcv_mlp <- bestcv4cls2(
  wkflow = wk_mlp,
  tuneresult = tune_mlp,
  hpbest = hpbest_mlp,
  yname = "Survival.months",
  modelname = "mlp",
  v = 10,
  positivelevel = yourpositivelevel
)
evalcv_mlp$cvroc
evalcv_mlp$cvpr
evalcv_mlp$evalcv


evalcv_svm <- bestcv4cls2(
  wkflow = wk_svm,
  tuneresult = tune_svm,
  hpbest = hpbest_svm,
  yname = "Survival.months",
  modelname = "svm",
  v = 10,
  positivelevel = yourpositivelevel
)
evalcv_svm$cvroc
evalcv_svm$cvpr
evalcv_svm$evalcv



evalcv_knn <- bestcv4cls2(
  wkflow = wk_knn,
  tuneresult = tune_knn,
  hpbest = hpbest_knn,
  yname = "Survival.months",
  modelname = "knn",
  v = 10,
  positivelevel = yourpositivelevel
)
evalcv_knn$cvroc
evalcv_knn$cvpr
evalcv_knn$evalcv


cv_logistic <-
  wk_logistic %>%
  fit_resamples(
    folds,
    metrics = metric_set(yardstick::accuracy,
```

```
                          yardstick::roc_auc,
                          yardstick::pr_auc),
    control = control_resamples(save_pred = T,
                                verbose = T,
                                event_level = "second",
                                parallel_over = "everything",
                                save_workflow = T)
  )
cv_logistic


evalcv_logistic <- list()

metrictemp <- metric_set(yardstick::roc_auc, yardstick::pr_
   auc)
evalcv_logistic$evalcv <-
  collect_predictions(cv_logistic) %>%
  group_by(id) %>%
  metrictemp(Survival.months, .pred_Yes, event_level = "
     second") %>%
  group_by(.metric) %>%
  mutate(model = "logistic",
         mean = mean(.estimate),
         sd = sd(.estimate)/sqrt(length(folds$splits)))
evalcv_logistic$evalcv



evalcv_logistic$cvroc <-
  collect_predictions(cv_logistic) %>%
  group_by(id) %>%
  roc_curve(Survival.months, .pred_Yes, event_level = "second
     ") %>%
  ungroup() %>%
  left_join(evalcv_logistic$evalcv %>% filter(.metric == "roc
     _auc"),
            by = "id") %>%
  mutate(idAUC = paste(id, " ROCAUC:", round(.estimate, 4)),
         idAUC = forcats::as_factor(idAUC)) %>%
  ggplot(aes(x = 1-specificity, y = sensitivity, color =
     idAUC)) +
  geom_path(linewidth = 1) +
  geom_abline(linetype = "dashed") +
  scale_x_continuous(expand = c(0,0)) +
  scale_y_continuous(expand = c(0,0)) +
  labs(color = "") +
  theme_bw() +
  theme(legend.position = c(1,0),
```

```
        legend.justification = c(1,0),
        legend.background = element_blank(),
        legend.key = element_blank())
evalcv_logistic$cvroc

evalcv_logistic$cvpr <-
  collect_predictions(cv_logistic) %>%
  group_by(id) %>%
  pr_curve(Survival.months, .pred_Yes, event_level = "second"
     ) %>%
  ungroup() %>%
  left_join(evalcv_logistic$evalcv %>% filter(.metric == "pr_
     auc"),
            by = "id") %>%
  mutate(idAUC = paste(id, " PRAUC:", round(.estimate, 4)),
         idAUC = forcats::as_factor(idAUC)) %>%
  ggplot(aes(x = recall, y = precision, color = idAUC)) +
  geom_path(linewidth = 1) +
  geom_abline(linetype = "dashed", intercept = 1, slope = -1)
     +
  scale_x_continuous(expand = c(0,0)) +
  scale_y_continuous(expand = c(0,0), limits = c(0, 1)) +
  labs(color = "") +
  theme_bw() +
  theme(legend.position = c(0,0),
        legend.justification = c(0,0),
        legend.background = element_blank(),
        legend.key = element_blank())
evalcv_logistic$cvpr










#14.
predtrain_dt <- valuateBinaryClassificationModel(
  model = final_dt,
  dataset = traindata,
  yname = "Survival.months",
  modelname = "DT",
  datasetname = "traindata",
  cutoff = "yueden",
  positivelevel = yourpositivelevel,
```

```
  negativelevel = yournegativelevel
)
predtrain_dt$prediction
predtrain_dt$rocresult
predtrain_dt$rocplot
predtrain_dt$prresult
predtrain_dt$prplot
predtrain_dt$cmresult
predtrain_dt$cmplot
predtrain_dt$metrics
predtrain_dt$diycutoff
predtrain_dt$ksplot
predtrain_dt$dcaplot

pROC::auc(predtrain_dt$proc)
pROC::ci.auc(predtrain_dt$proc)


predtest_dt <- valuateBinaryClassificationModel(
  model = final_dt,
  dataset = testdata,
  yname = "Survival.months",
  modelname = "DT",
  datasetname = "testdata",
  cutoff = predtrain_dt$diycutoff,
  positivelevel = yourpositivelevel,
  negativelevel = yournegativelevel
)
predtest_dt$prediction
predtest_dt$rocresult
predtest_dt$rocplot
predtest_dt$prresult
predtest_dt$prplot
predtest_dt$cmresult
predtest_dt$cmplot
predtest_dt$metrics
predtest_dt$diycutoff
predtest_dt$ksplot
predtest_dt$dcaplot

pROC::auc(predtest_dt$proc)
pROC::ci.auc(predtest_dt$proc)


predtrain_dt$rocresult %>%
  bind_rows(predtest_dt$rocresult) %>%
  mutate(dataAUC = paste(data, " ROCAUC:", round(ROCAUC, 4)),
         dataAUC = forcats::as_factor(dataAUC)) %>%
```

```
  ggplot(aes(x = 1-specificity,
             y = sensitivity,
             color = dataAUC)) +
  geom_path(linewidth = 1) +
  geom_abline(linetype = "dashed") +
  scale_x_continuous(expand = c(0,0)) +
  scale_y_continuous(expand = c(0,0)) +
  labs(color = "") +
  theme_bw() +
  theme(legend.position = c(1,0),
        legend.justification = c(1,0),
        legend.background = element_blank(),
        legend.key = element_blank())

predtrain_dt$prresult %>%
  bind_rows(predtest_dt$prresult) %>%
  mutate(dataAUC = paste(data, " PRAUC:", round(PRAUC, 4)),
         dataAUC = forcats::as_factor(dataAUC)) %>%
  ggplot(aes(x = recall,
             y = precision,
             color = dataAUC)) +
  geom_path(linewidth = 1) +
  geom_abline(linetype = "dashed", slope = -1, intercept = 1)
    +
  scale_x_continuous(expand = c(0,0)) +
  scale_y_continuous(expand = c(0,0), limits = c(0, 1)) +
  labs(color = "") +
  theme_bw() +
  theme(legend.position = c(0,0),
        legend.justification = c(0,0),
        legend.background = element_blank(),
        legend.key = element_blank())





predtrain_rf <- valuateBinaryClassificationModel(
  model = final_rf,
  dataset = traindata,
  yname = "Survival.months",
  modelname = "rf",
  datasetname = "traindata",
  cutoff = "yueden",
  positivelevel = yourpositivelevel,
  negativelevel = yournegativelevel
)
predtrain_rf$prediction
```

```
predtrain_rf$rocresult
predtrain_rf$rocplot
predtrain_rf$prresult
predtrain_rf$prplot
predtrain_rf$cmresult
predtrain_rf$cmplot
predtrain_rf$metrics
predtrain_rf$diycutoff
predtrain_rf$ksplot
predtrain_rf$dcaplot

pROC::auc(predtrain_rf$proc)
pROC::ci.auc(predtrain_rf$proc)


predtest_rf <- valuateBinaryClassificationModel(
  model = final_rf,
  dataset = testdata,
  yname = "Survival.months",
  modelname = "rf",
  datasetname = "testdata",
  cutoff = predtrain_rf$diycutoff,
  positivelevel = yourpositivelevel,
  negativelevel = yournegativelevel
)
predtest_rf$prediction
predtest_rf$rocresult
predtest_rf$rocplot
predtest_rf$prresult
predtest_rf$prplot
predtest_rf$cmresult
predtest_rf$cmplot
predtest_rf$metrics
predtest_rf$diycutoff
predtest_rf$ksplot
predtest_rf$dcaplot

pROC::auc(predtest_rf$proc)
pROC::ci.auc(predtest_rf$proc)



predtrain_rf$rocresult %>%
  bind_rows(predtest_rf$rocresult) %>%
  mutate(dataAUC = paste(data, " ROCAUC:", round(ROCAUC, 4)),
         dataAUC = forcats::as_factor(dataAUC)) %>%
  ggplot(aes(x = 1-specificity,
             y = sensitivity,
```

```r
              color = dataAUC)) +
  geom_path(linewidth = 1) +
  geom_abline(linetype = "dashed") +
  scale_x_continuous(expand = c(0,0)) +
  scale_y_continuous(expand = c(0,0)) +
  labs(color = "") +
  theme_bw() +
  theme(legend.position = c(1,0),
        legend.justification = c(1,0),
        legend.background = element_blank(),
        legend.key = element_blank())

predtrain_rf$prresult %>%
  bind_rows(predtest_rf$prresult) %>%
  mutate(dataAUC = paste(data, " PRAUC:", round(PRAUC, 4)),
         dataAUC = forcats::as_factor(dataAUC)) %>%
  ggplot(aes(x = recall,
              y = precision,
              color = dataAUC)) +
  geom_path(linewidth = 1) +
  geom_abline(linetype = "dashed", slope = -1, intercept = 1)
     +
  scale_x_continuous(expand = c(0,0)) +
  scale_y_continuous(expand = c(0,0), limits = c(0, 1)) +
  labs(color = "") +
  theme_bw() +
  theme(legend.position = c(0,0),
        legend.justification = c(0,0),
        legend.background = element_blank(),
        legend.key = element_blank())





predtrain_xgboost <- valuateBinaryClassificationModel(
  model = final_xgboost,
  dataset = traindata,
  yname = "Survival.months",
  modelname = "xgboost",
  datasetname = "traindata",
  cutoff = "yueden",
  positivelevel = yourpositivelevel,
  negativelevel = yournegativelevel
)
predtrain_xgboost$prediction
predtrain_xgboost$rocresult
predtrain_xgboost$rocplot
```

```
predtrain_xgboost$prresult
predtrain_xgboost$prplot
predtrain_xgboost$cmresult
predtrain_xgboost$cmplot
predtrain_xgboost$metrics
predtrain_xgboost$diycutoff
predtrain_xgboost$ksplot
predtrain_xgboost$dcaplot

pROC::auc(predtrain_xgboost$proc)
pROC::ci.auc(predtrain_xgboost$proc)

#
predtest_xgboost <- valuateBinaryClassificationModel(
  model = final_xgboost,
  dataset = testdata,
  yname = "Survival.months",
  modelname = "xgboost",
  datasetname = "testdata",
  cutoff = predtrain_xgboost$diycutoff,
  positivelevel = yourpositivelevel,
  negativelevel = yournegativelevel
)
predtest_xgboost$prediction
predtest_xgboost$rocresult
predtest_xgboost$rocplot
predtest_xgboost$prresult
predtest_xgboost$prplot
predtest_xgboost$cmresult
predtest_xgboost$cmplot
predtest_xgboost$metrics
predtest_xgboost$diycutoff
predtest_xgboost$ksplot
predtest_xgboost$dcaplot

pROC::auc(predtest_xgboost$proc)
pROC::ci.auc(predtest_xgboost$proc)


#
predtrain_xgboost$rocresult %>%
  bind_rows(predtest_xgboost$rocresult) %>%
  mutate(dataAUC = paste(data, " ROCAUC:", round(ROCAUC, 4)),
         dataAUC = forcats::as_factor(dataAUC)) %>%
  ggplot(aes(x = 1-specificity,
             y = sensitivity,
             color = dataAUC)) +
  geom_path(linewidth = 1) +
```

```
  geom_abline(linetype = "dashed") +
  scale_x_continuous(expand = c(0,0)) +
  scale_y_continuous(expand = c(0,0)) +
  labs(color = "") +
  theme_bw() +
  theme(legend.position = c(1,0),
        legend.justification = c(1,0),
        legend.background = element_blank(),
        legend.key = element_blank())

predtrain_xgboost$prresult %>%
  bind_rows(predtest_xgboost$prresult) %>%
  mutate(dataAUC = paste(data, " PRAUC:", round(PRAUC, 4)),
         dataAUC = forcats::as_factor(dataAUC)) %>%
  ggplot(aes(x = recall,
             y = precision,
             color = dataAUC)) +
  geom_path(linewidth = 1) +
  geom_abline(linetype = "dashed", slope = -1, intercept = 1)
    +
  scale_x_continuous(expand = c(0,0)) +
  scale_y_continuous(expand = c(0,0), limits = c(0, 1)) +
  labs(color = "") +
  theme_bw() +
  theme(legend.position = c(0,0),
        legend.justification = c(0,0),
        legend.background = element_blank(),
        legend.key = element_blank())




predtrain_lightgbm <- valuateBinaryClassificationModel(
  model = final_lightgbm,
  dataset = traindata,
  yname = "Survival.months",
  modelname = "lightgbm",
  datasetname = "traindata",
  cutoff = "yueden",
  positivelevel = yourpositivelevel,
  negativelevel = yournegativelevel
)
predtrain_lightgbm$prediction
predtrain_lightgbm$rocresult
predtrain_lightgbm$rocplot
predtrain_lightgbm$prresult
predtrain_lightgbm$prplot
predtrain_lightgbm$cmresult
```

```
predtrain_lightgbm$cmplot
predtrain_lightgbm$metrics
predtrain_lightgbm$diycutoff
predtrain_lightgbm$ksplot
predtrain_lightgbm$dcaplot

pROC::auc(predtrain_lightgbm$proc)
pROC::ci.auc(predtrain_lightgbm$proc)

#
predtest_lightgbm <- valuateBinaryClassificationModel(
  model = final_lightgbm,
  dataset = testdata,
  yname = "Survival.months",
  modelname = "lightgbm",
  datasetname = "testdata",
  cutoff = predtrain_lightgbm$diycutoff,
  positivelevel = yourpositivelevel,
  negativelevel = yournegativelevel
)
predtest_lightgbm$prediction
predtest_lightgbm$rocresult
predtest_lightgbm$rocplot
predtest_lightgbm$prresult
predtest_lightgbm$prplot
predtest_lightgbm$cmresult
predtest_lightgbm$cmplot
predtest_lightgbm$metrics
predtest_lightgbm$diycutoff
predtest_lightgbm$ksplot
predtest_lightgbm$dcaplot

pROC::auc(predtest_lightgbm$proc)
pROC::ci.auc(predtest_lightgbm$proc)


#
predtrain_lightgbm$rocresult %>%
  bind_rows(predtest_lightgbm$rocresult) %>%
  mutate(dataAUC = paste(data, " ROCAUC:", round(ROCAUC, 4)),
         dataAUC = forcats::as_factor(dataAUC)) %>%
  ggplot(aes(x = 1-specificity,
             y = sensitivity,
             color = dataAUC)) +
  geom_path(linewidth = 1) +
  geom_abline(linetype = "dashed") +
  scale_x_continuous(expand = c(0,0)) +
  scale_y_continuous(expand = c(0,0)) +
```

```
  labs(color = "") +
  theme_bw() +
  theme(legend.position = c(1,0),
        legend.justification = c(1,0),
        legend.background = element_blank(),
        legend.key = element_blank())

predtrain_lightgbm$prresult %>%
  bind_rows(predtest_lightgbm$prresult) %>%
  mutate(dataAUC = paste(data, " PRAUC:", round(PRAUC, 4)),
         dataAUC = forcats::as_factor(dataAUC)) %>%
  ggplot(aes(x = recall,
             y = precision,
             color = dataAUC)) +
  geom_path(linewidth = 1) +
  geom_abline(linetype = "dashed", slope = -1, intercept = 1)
    +
  scale_x_continuous(expand = c(0,0)) +
  scale_y_continuous(expand = c(0,0), limits = c(0, 1)) +
  labs(color = "") +
  theme_bw() +
  theme(legend.position = c(0,0),
        legend.justification = c(0,0),
        legend.background = element_blank(),
        legend.key = element_blank())





predtrain_enet <- valuateBinaryClassificationModel(
  model = final_enet,
  dataset = traindata,
  yname = "Survival.months",
  modelname = "enet",
  datasetname = "traindata",
  cutoff = "yueden",
  positivelevel = yourpositivelevel,
  negativelevel = yournegativelevel
)
predtrain_enet$prediction
predtrain_enet$rocresult
predtrain_enet$rocplot
predtrain_enet$prresult
predtrain_enet$prplot
predtrain_enet$cmresult
predtrain_enet$cmplot
predtrain_enet$metrics
```

```r
predtrain_enet$diycutoff
predtrain_enet$ksplot
predtrain_enet$dcaplot

pROC::auc(predtrain_enet$proc)
pROC::ci.auc(predtrain_enet$proc)
#
  dataset = testdata,
  yname = "Survival.months",
  modelname = "enet",
  datasetname = "testdata",
  cutoff = predtrain_enet$diycutoff,
  positivelevel = yourpositivelevel,
  negativelevel = yournegativelevel
)
predtest_enet$prediction
predtest_enet$rocresult
predtest_enet$rocplot
predtest_enet$prresult
predtest_enet$prplot
predtest_enet$cmresult
predtest_enet$cmplot
predtest_enet$metrics
predtest_enet$diycutoff
predtest_enet$ksplot
predtest_enet$dcaplot

pROC::auc(predtest_enet$proc)
pROC::ci.auc(predtest_enet$proc)


#
predtrain_enet$rocresult %>%
  bind_rows(predtest_enet$rocresult) %>%
  mutate(dataAUC = paste(data, " ROCAUC:", round(ROCAUC, 4)),
         dataAUC = forcats::as_factor(dataAUC)) %>%
  ggplot(aes(x = 1-specificity,
             y = sensitivity,
             color = dataAUC)) +
  geom_path(linewidth = 1) +
  geom_abline(linetype = "dashed") +
  scale_x_continuous(expand = c(0,0)) +
  scale_y_continuous(expand = c(0,0)) +
  labs(color = "") +
  theme_bw() +
  theme(legend.position = c(1,0),
        legend.justification = c(1,0),
        legend.background = element_blank(),
```

```
        legend.key = element_blank())

predtrain_enet$prresult %>%
  bind_rows(predtest_enet$prresult) %>%
  mutate(dataAUC = paste(data, " PRAUC:", round(PRAUC, 4)),
         dataAUC = forcats::as_factor(dataAUC)) %>%
  ggplot(aes(x = recall,
             y = precision,
             color = dataAUC)) +
  geom_path(linewidth = 1) +
  geom_abline(linetype = "dashed", slope = -1, intercept = 1)
      +
  scale_x_continuous(expand = c(0,0)) +
  scale_y_continuous(expand = c(0,0), limits = c(0, 1)) +
  labs(color = "") +
  theme_bw() +
  theme(legend.position = c(0,0),
        legend.justification = c(0,0),
        legend.background = element_blank(),
        legend.key = element_blank())





predtrain_mlp <- valuateBinaryClassificationModel(
  model = final_mlp,
  dataset = traindata,
  yname = "Survival.months",
  modelname = "mlp",
  datasetname = "traindata",
  cutoff = "yueden",
  positivelevel = yourpositivelevel,
  negativelevel = yournegativelevel
)
predtrain_mlp$prediction
predtrain_mlp$rocresult
predtrain_mlp$rocplot
predtrain_mlp$prresult
predtrain_mlp$prplot
predtrain_mlp$cmresult
predtrain_mlp$cmplot
predtrain_mlp$metrics
predtrain_mlp$diycutoff
predtrain_mlp$ksplot
predtrain_mlp$dcaplot

pROC::auc(predtrain_mlp$proc)
```

```
pROC::ci.auc(predtrain_mlp$proc)

#
predtest_mlp <- valuateBinaryClassificationModel(
  model = final_mlp,
  dataset = testdata,
  yname = "Survival.months",
  modelname = "mlp",
  datasetname = "testdata",
  cutoff = predtrain_mlp$diycutoff,
  positivelevel = yourpositivelevel,
  negativelevel = yournegativelevel
)
predtest_mlp$prediction
predtest_mlp$rocresult
predtest_mlp$rocplot
predtest_mlp$prresult
predtest_mlp$prplot
predtest_mlp$cmresult
predtest_mlp$cmplot
predtest_mlp$metrics
predtest_mlp$diycutoff
predtest_mlp$ksplot
predtest_mlp$dcaplot

pROC::auc(predtest_mlp$proc)
pROC::ci.auc(predtest_mlp$proc)


#
predtrain_mlp$rocresult %>%
  bind_rows(predtest_mlp$rocresult) %>%
  mutate(dataAUC = paste(data, " ROCAUC:", round(ROCAUC, 4)),
         dataAUC = forcats::as_factor(dataAUC)) %>%
  ggplot(aes(x = 1-specificity,
             y = sensitivity,
             color = dataAUC)) +
  geom_path(linewidth = 1) +
  geom_abline(linetype = "dashed") +
  scale_x_continuous(expand = c(0,0)) +
  scale_y_continuous(expand = c(0,0)) +
  labs(color = "") +
  theme_bw() +
  theme(legend.position = c(1,0),
        legend.justification = c(1,0),
        legend.background = element_blank(),
        legend.key = element_blank())

```

```
predtrain_mlp$prresult %>%
  bind_rows(predtest_mlp$prresult) %>%
  mutate(dataAUC = paste(data, " PRAUC:", round(PRAUC, 4)),
         dataAUC = forcats::as_factor(dataAUC)) %>%
  ggplot(aes(x = recall,
             y = precision,
             color = dataAUC)) +
  geom_path(linewidth = 1) +
  geom_abline(linetype = "dashed", slope = -1, intercept = 1)
    +
  scale_x_continuous(expand = c(0,0)) +
  scale_y_continuous(expand = c(0,0), limits = c(0, 1)) +
  labs(color = "") +
  theme_bw() +
  theme(legend.position = c(0,0),
        legend.justification = c(0,0),
        legend.background = element_blank(),
        legend.key = element_blank())




predtrain_svm <- valuateBinaryClassificationModel(
  model = final_svm,
  dataset = traindata,
  yname = "Survival.months",
  modelname = "svm",
  datasetname = "traindata",
  cutoff = "yueden",
  positivelevel = yourpositivelevel,
  negativelevel = yournegativelevel
)
predtrain_svm$prediction
predtrain_svm$rocresult
predtrain_svm$rocplot
predtrain_svm$prresult
predtrain_svm$prplot
predtrain_svm$cmresult
predtrain_svm$cmplot
predtrain_svm$metrics
predtrain_svm$diycutoff
predtrain_svm$ksplot
predtrain_svm$dcaplot

pROC::auc(predtrain_svm$proc)
pROC::ci.auc(predtrain_svm$proc)

#
```

```
predtest_svm <- valuateBinaryClassificationModel(
  model = final_svm,
  dataset = testdata,
  yname = "Survival.months",
  modelname = "svm",
  datasetname = "testdata",
  cutoff = predtrain_svm$diycutoff,
  positivelevel = yourpositivelevel,
  negativelevel = yournegativelevel
)
predtest_svm$prediction
predtest_svm$rocresult
predtest_svm$rocplot
predtest_svm$prresult
predtest_svm$prplot
predtest_svm$cmresult
predtest_svm$cmplot
predtest_svm$metrics
predtest_svm$diycutoff
predtest_svm$ksplot
predtest_svm$dcaplot

pROC::auc(predtest_svm$proc)
pROC::ci.auc(predtest_svm$proc)


#
predtrain_svm$rocresult %>%
  bind_rows(predtest_svm$rocresult) %>%
  mutate(dataAUC = paste(data, " ROCAUC:", round(ROCAUC, 4)),
         dataAUC = forcats::as_factor(dataAUC)) %>%
  ggplot(aes(x = 1-specificity,
             y = sensitivity,
             color = dataAUC)) +
  geom_path(linewidth = 1) +
  geom_abline(linetype = "dashed") +
  scale_x_continuous(expand = c(0,0)) +
  scale_y_continuous(expand = c(0,0)) +
  labs(color = "") +
  theme_bw() +
  theme(legend.position = c(1,0),
        legend.justification = c(1,0),
        legend.background = element_blank(),
        legend.key = element_blank())

predtrain_svm$prresult %>%
  bind_rows(predtest_svm$prresult) %>%
  mutate(dataAUC = paste(data, " PRAUC:", round(PRAUC, 4)),
```

```
          dataAUC = forcats::as_factor(dataAUC)) %>%
  ggplot(aes(x = recall,
              y = precision,
              color = dataAUC)) +
  geom_path(linewidth = 1) +
  geom_abline(linetype = "dashed", slope = -1, intercept = 1) +
  scale_x_continuous(expand = c(0,0)) +
  scale_y_continuous(expand = c(0,0), limits = c(0, 1)) +
  labs(color = "") +
  theme_bw() +
  theme(legend.position = c(0,0),
        legend.justification = c(0,0),
        legend.background = element_blank(),
        legend.key = element_blank())



predtrain_knn <- valuateBinaryClassificationModel(
  model = final_knn,
  dataset = traindata,
  yname = "Survival.months",
  modelname = "knn",
  datasetname = "traindata",
  cutoff = "yueden",
  positivelevel = yourpositivelevel,
  negativelevel = yournegativelevel
)
predtrain_knn$prediction
predtrain_knn$rocresult
predtrain_knn$rocplot
predtrain_knn$prresult
predtrain_knn$prplot
predtrain_knn$cmresult
predtrain_knn$cmplot
predtrain_knn$metrics
predtrain_knn$diycutoff
predtrain_knn$ksplot
predtrain_knn$dcaplot

pROC::auc(predtrain_knn$proc)
pROC::ci.auc(predtrain_knn$proc)

#  valuateBinaryClassificationModel(
  model = final_knn,
  dataset = testdata,
  yname = "Survival.months",
  modelname = "knn",
```

```
  datasetname = "testdata",
  cutoff = predtrain_knn$diycutoff,
  positivelevel = yourpositivelevel,
  negativelevel = yournegativelevel
)
predtest_knn$prediction
predtest_knn$rocresult
predtest_knn$rocplot
predtest_knn$prresult
predtest_knn$prplot
predtest_knn$cmresult
predtest_knn$cmplot
predtest_knn$metrics
predtest_knn$diycutoff
predtest_knn$ksplot
predtest_knn$dcaplot

pROC::auc(predtest_knn$proc)
pROC::ci.auc(predtest_knn$proc)


#
predtrain_knn$rocresult %>%
  bind_rows(predtest_knn$rocresult) %>%
  mutate(dataAUC = paste(data, " ROCAUC:", round(ROCAUC, 4)),
         dataAUC = forcats::as_factor(dataAUC)) %>%
  ggplot(aes(x = 1-specificity,
             y = sensitivity,
             color = dataAUC)) +
  geom_path(linewidth = 1) +
  geom_abline(linetype = "dashed") +
  scale_x_continuous(expand = c(0,0)) +
  scale_y_continuous(expand = c(0,0)) +
  labs(color = "") +
  theme_bw() +
  theme(legend.position = c(1,0),
        legend.justification = c(1,0),
        legend.background = element_blank(),
        legend.key = element_blank())

predtrain_knn$prresult %>%
  bind_rows(predtest_knn$prresult) %>%
  mutate(dataAUC = paste(data, " PRAUC:", round(PRAUC, 4)),
         dataAUC = forcats::as_factor(dataAUC)) %>%
  ggplot(aes(x = recall,
             y = precision,
             color = dataAUC)) +
  geom_path(linewidth = 1) +
```

```
  geom_abline(linetype = "dashed", slope = -1, intercept = 1)
      +
  scale_x_continuous(expand = c(0,0)) +
  scale_y_continuous(expand = c(0,0), limits = c(0, 1)) +
  labs(color = "") +
  theme_bw() +
  theme(legend.position = c(0,0),
        legend.justification = c(0,0),
        legend.background = element_blank(),
        legend.key = element_blank())



predtrain_logistic <- valuateBinaryClassificationModel(
  model = final_logistic,
  dataset = traindata,
  yname = "Survival.months",
  modelname = "Logistic",
  datasetname = "traindata",
  cutoff = "yueden",
  positivelevel = yourpositivelevel,
  negativelevel = yournegativelevel
)
predtrain_logistic$prediction
predtrain_logistic$predprobplot
predtrain_logistic$rocresult
predtrain_logistic$rocplot
predtrain_logistic$prresult
predtrain_logistic$prplot
predtrain_logistic$cmresult
predtrain_logistic$cmplot
predtrain_logistic$metrics
predtrain_logistic$diycutoff
predtrain_logistic$ksplot
predtrain_logistic$dcaplot

pROC::auc(predtrain_logistic$proc)
pROC::ci.auc(predtrain_logistic$proc)

#
predtest_logistic <- valuateBinaryClassificationModel(
  model = final_logistic,
  dataset = testdata,
  yname = "Survival.months",
  modelname = "Logistic",
  datasetname = "testdata",
  cutoff = predtrain_logistic$diycutoff,
  positivelevel = yourpositivelevel,
```

```
  negativelevel = yournegativelevel
)
predtest_logistic$prediction
predtest_logistic$predprobplot
predtest_logistic$rocresult
predtest_logistic$rocplot
predtest_logistic$prresult
predtest_logistic$prplot
predtest_logistic$cmresult
predtest_logistic$cmplot
predtest_logistic$metrics
predtest_logistic$diycutoff
predtest_logistic$ksplot
predtest_logistic$dcaplot

pROC::auc(predtest_logistic$proc)
pROC::ci.auc(predtest_logistic$proc)
pROC::roc.test(predtrain_logistic$proc, predtest_logistic$
   proc)

#
predtrain_logistic$rocresult %>%
  bind_rows(predtest_logistic$rocresult) %>%
  mutate(dataAUC = paste(data, " ROCAUC:", round(ROCAUC, 4)),
         dataAUC = forcats::as_factor(dataAUC)) %>%
  ggplot(aes(x = 1-specificity,
             y = sensitivity,
             color = dataAUC)) +
  geom_path(linewidth = 1) +
  geom_abline(linetype = "dashed") +
  scale_x_continuous(expand = c(0,0)) +
  scale_y_continuous(expand = c(0,0)) +
  labs(color = "") +
  theme_bw() +
  theme(legend.position = c(1,0),
        legend.justification = c(1,0),
        legend.background = element_blank(),
        legend.key = element_blank())

predtrain_logistic$prresult %>%
  bind_rows(predtest_logistic$prresult) %>%
  mutate(dataAUC = paste(data, " PRAUC:", round(PRAUC, 4)),
         dataAUC = forcats::as_factor(dataAUC)) %>%
  ggplot(aes(x = recall,
             y = precision,
             color = dataAUC)) +
  geom_path(linewidth = 1) +
  geom_abline(linetype = "dashed", slope = -1, intercept = 1)
```

```
      +
  scale_x_continuous(expand = c(0,0)) +
  scale_y_continuous(expand = c(0,0), limits = c(0, 1)) +
  labs(color = "") +
  theme_bw() +
  theme(legend.position = c(0,0),
        legend.justification = c(0,0),
        legend.background = element_blank(),
        legend.key = element_blank())
#
predtrain_logistic$metrics %>%
  bind_rows(predtest_logistic$metrics) %>%
  dplyr::select(-.estimator) %>%
  pivot_wider(names_from = .metric, values_from = .estimate)






#15
save(datarecipe_dt,
     model_dt,
     wk_dt,
     hpgrid_dt,
     tune_dt,
     predtrain_dt,
     predtest_dt,
     evalcv_dt,
     file = ".\\MLresult\\MLdt.RData")


save(datarecipe_rf,
     model_rf,
     wk_rf,
     hpgrid_rf,
     tune_rf,
     predtrain_rf,
     predtest_rf,
     evalcv_rf,
     file = ".\\MLresult\\MLrf.RData")


save(datarecipe_xgboost,
     model_xgboost,
     wk_xgboost,
     hpgrid_xgboost,
     tune_xgboost,
```

```
      predtrain_xgboost,
      predtest_xgboost,
      evalcv_xgboost,
      file = ".\\MLresult\\MLxgboost.RData")


save(datarecipe_enet,
      model_enet,
      wk_enet,
      hpgrid_enet,
      tune_enet,
      predtrain_enet,
      predtest_enet,
      evalcv_enet,
      file = ".\\MLresult\\MLenet.RData")


save(datarecipe_svm,
      model_svm,
      wk_svm,
      hpgrid_svm,
      tune_svm,
      predtrain_svm,
      predtest_svm,
      evalcv_svm,
      file = ".\\MLresult\\MLsvm.RData")


save(datarecipe_mlp,
      model_mlp,
      wk_mlp,
      hpgrid_mlp,
      tune_mlp,
      predtrain_mlp,
      predtest_mlp,
      evalcv_mlp,
      file = ".\\MLresult\\MLmlp.RData")



save(datarecipe_lightgbm,
      model_lightgbm,
      wk_lightgbm,
      hpgrid_lightgbm,
      tune_lightgbm,
      predtrain_lightgbm,
      predtest_lightgbm,
      evalcv_lightgbm,
```

```r
     file = ".\\MLresult\\MLlightgbm.RData")



save(datarecipe_knn,
     model_knn,
     wk_knn,
     hpgrid_knn,
     tune_knn,
     predtrain_knn,
     predtest_knn,
     evalcv_knn,
     file = ".\\MLresult\\MLknn.RData")


save(datarecipe_logistic,
     model_logistic,
     wk_logistic,
     cv_logistic,
     predtrain_logistic,
     predtest_logistic,
     evalcv_logistic,
     file = ".\\MLresult\\MLlogistic.RData")

















#16
#
evalfiles <- list.files(".\\MLresult\\", full.names = T)
lapply(evalfiles, load, .GlobalEnv)

#
nmodels <- 9

```

```
#
cols4model <- c("#E41A1C",  #
                "#377EB8",  #
                "#4DAF4A",  #
                "#984EA3",  #
                "#FF7F00",  #
                "#FFFF33",  #
                "#A65628",  #
                "#F781BF",  #
                "#999999")  #


#
predtest_dt$metrics
eval <- bind_rows(
  lapply(list(predtest_logistic, predtest_dt, predtest_enet,
              predtest_knn, predtest_lightgbm, predtest_rf,
              predtest_xgboost, predtest_svm, predtest_mlp),
         "[[",
         "metrics")
) %>%
  mutate(model = forcats::as_factor(model))
eval

#
eval2 <- eval %>%
  dplyr::select(-.estimator) %>%
  pivot_wider(names_from = .metric, values_from = .estimate)
eval2

#
predtest <- bind_rows(
  lapply(list(predtest_logistic, predtest_dt, predtest_enet,
              predtest_knn, predtest_lightgbm, predtest_rf,
              predtest_xgboost, predtest_svm, predtest_mlp),
         "[[",
         "prediction")
) %>%
  mutate(model = forcats::as_factor(model))
predtest

# ROC
predtest %>%
  group_by(model) %>%
  roc_curve(.obs, .pred_Yes, event_level = "second") %>%
  left_join(eval2[, c("model", "roc_auc")]) %>%
  mutate(modelauc = paste0(model,
                           ", ROCAUC=", round(roc_auc, 4)),
```

```r
         modelauc = forcats::as_factor(modelauc)) %>%
  ggplot(aes(x = 1-specificity, y = sensitivity, color =
     modelauc)) +
  geom_path(linewidth = 1) +
  geom_abline(linetype = "dashed") +
  scale_color_manual(values = cols4model) +
  scale_x_continuous(limits = c(0, 1), expand = c(0, 0)) +
  scale_y_continuous(limits = c(0, 1), expand = c(0, 0)) +
  labs(color = "", title = paste0("ROCs on testdata")) +
  theme_bw() +
  theme(legend.position = c(1,0),
        legend.justification = c(1,0),
        legend.background = element_blank(),
        legend.key = element_blank())

# PRAUC
predtest %>%
  group_by(model) %>%
  pr_curve(.obs, .pred_Yes, event_level = "second") %>%
  left_join(eval2[, c("model", "pr_auc")]) %>%
  mutate(modelauc = paste0(model,
                           ", PRAUC=", round(pr_auc, 4)),
         modelauc = forcats::as_factor(modelauc)) %>%
  ggplot(aes(x = recall, y = precision, color = modelauc)) +
  geom_path(linewidth = 1) +
  geom_abline(linetype = "dashed", slope = -1, intercept = 1)
    +
  scale_color_manual(values = cols4model) +
  scale_x_continuous(limits = c(0, 1), expand = c(0, 0)) +
  scale_y_continuous(limits = c(0, 1), expand = c(0, 0)) +
  labs(color = "", title = paste0("PRs on testdata")) +
  theme_bw() +
  theme(legend.position = c(0,0),
        legend.justification = c(0,0),
        legend.background = element_blank(),
        legend.key = element_blank())

##############################################################

#
predtest2 <- predtest %>%
  dplyr::select(-.pred_No) %>%
  mutate(id = rep(1:nrow(predtest_logistic$prediction),
                  length(unique(predtest$model)))) %>%
  pivot_wider(id_cols = c(id, .obs),
              names_from = model,
              values_from = .pred_Yes) %>%
  dplyr::select(id, .obs, sort(unique(predtest$model)))
```

```
predtest2

###############################################################


#
library(probably)

#
predtest %>%
  cal_plot_breaks(.obs, .pred_Yes, event_level = "second",
     num_breaks = 5, .by = model) +
  scale_color_manual(values = cols4model) +
  theme_bw() +
  theme(legend.position = "none")

#
predtest %>%
  cal_plot_windowed(.obs, .pred_Yes, event_level = "second",
     window_size = 0.5, .by = model) +
  scale_color_manual(values = cols4model) +
  theme_bw() +
  theme(legend.position = "none")

#
bs <- predtest %>%
  group_by(model) %>%
  yardstick::brier_class(.obs, .pred_No) %>%
  mutate(meanpred = 0.8, meanobs = 0.25, text = paste0("BS: "
     , round(.estimate, 3)))

#
predtest %>%
  cal_plot_windowed(.obs, .pred_Yes, event_level = "second",
     window_size = 0.5, .by = model) +
  geom_text(bs, mapping = aes(x = meanpred, y = meanobs,
     label = text)) +
  scale_color_manual(values = cols4model) +
  theme_bw() +
  theme(legend.position = "none")



# DCA
dca_obj <- dcurves::dca(as.formula(
  paste0(".obs ~ ",
         paste(colnames(predtest2)[3:ncol(predtest2)],
               collapse = " + "))
```

```
),
data = predtest2,
thresholds = seq(0, 1, by = 0.01)
)
plot(dca_obj, smooth = T, span = 0.5) +
  scale_color_manual(values = c("black", "grey", cols4model)) +
  labs(title = "DCA on testdata")

##############################################################

#
evalcv <- bind_rows(
  lapply(list(evalcv_logistic, evalcv_dt, evalcv_enet,
              evalcv_knn, evalcv_lightgbm, evalcv_rf,
              evalcv_xgboost, evalcv_svm, evalcv_mlp),
         "[[",
         "evalcv")
) %>%
  mutate(
    model = forcats::as_factor(model),
    modelperf = paste0(model, "(", round(mean, 2)," ",
                       round(sd,2), ")")
  )
evalcv

# ROC
evalcvroc_max <-   evalcv %>%
  filter(.metric == "roc_auc") %>%
  group_by(id) %>%
  slice_max(.estimate)
evalcvroc_min <-   evalcv %>%
  filter(.metric == "roc_auc") %>%
  group_by(id) %>%
  slice_min(.estimate)
evalcv %>%
  filter(.metric == "roc_auc") %>%
  ggplot(aes(x = id, y = .estimate,
             group = modelperf, color = modelperf)) +
  geom_point() +
  geom_line() +
  ggrepel::geom_text_repel(evalcvroc_max,
                           mapping = aes(label = model),
                           nudge_y = 0.01,
                           show.legend = F) +
  ggrepel::geom_text_repel(evalcvroc_min,
                           mapping = aes(label = model),
                           nudge_y = -0.01,
```

```
                             show.legend = F) +
  scale_y_continuous(limits = c(0.5, 1)) +
  scale_color_manual(values = cols4model) +
  labs(x = "", y = "ROCAUC", color = "Model") +
  theme_bw()

# PR
evalcvpr_max <-   evalcv %>%
  filter(.metric == "pr_auc") %>%
  group_by(id) %>%
  slice_max(.estimate)
evalcvpr_min <-   evalcv %>%
  filter(.metric == "pr_auc") %>%
  group_by(id) %>%
  slice_min(.estimate)
evalcv %>%
  filter(.metric == "pr_auc") %>%
  ggplot(aes(x = id, y = .estimate,
             group = modelperf, color = modelperf)) +
  geom_point() +
  geom_line() +
  ggrepel::geom_text_repel(evalcvpr_max,
                           mapping = aes(label = model),
                           nudge_y = 0.01,
                           show.legend = F) +
  ggrepel::geom_text_repel(evalcvpr_min,
                           mapping = aes(label = model),
                           nudge_y = -0.01,
                           show.legend = F) +
  scale_y_continuous(limits = c(0.5, 1)) +
  scale_color_manual(values = cols4model) +
  labs(x = "", y = "prAUC", color = "Model") +
  theme_bw()

#
# ROC
evalcv %>%
  filter(.metric == "roc_auc") %>%
  group_by(model) %>%
  sample_n(size = 1) %>%
  ungroup() %>%
  ggplot(aes(x = model, y = mean, color = model)) +
  geom_point(size = 2, show.legend = F) +
  # geom_line(group = 1) +
  geom_errorbar(aes(ymin = mean-sd,
                    ymax = mean+sd),
                width = 0.1,
                linewidth = 1.2,
```

```
                show.legend = F) +
  scale_y_continuous(limits = c(0.7, 1)) +
  scale_color_manual(values = cols4model) +
  labs(y = "cv roc_auc") +
  theme_bw()









source("shap.R")


colnames(traindata)

traindatax <- traindata %>%
  dplyr::select(-Survival.months)

colnames(traindatax)


catvars <- colnames(traindatax)[sapply(traindatax, is.factor)]

catvars


convars <- setdiff(colnames(traindatax), catvars)

convars




shapresult <- generateShapVisualizations(
  finalmodel = final_xgboost,
  predfunc = function(model, newdata) {

    predict(model, newdata, type = "prob") %>%
      dplyr::select(ends_with(yourpositivelevel)) %>%
      pull()
  },
  datax = traindatax,
  datay = traindata$Survival.months,
```

```
  yname = "Survival.months",
  flname = catvars,
  lxname = convars
)

#                      SHAP
shapresult$shapvip
#
shapresult$shapvipplot

#
                                shapvizSHAP

shapley <- shapviz::shapviz(
  shapresult$shapley,  #    S   H   A   P
  X = traindatax,  #
  baseline = mean(predtrain_xgboost$prediction$.pred_Yes)  #
                                Yes
)
#                  1force
shapviz::sv_force(shapley, row_id = 1)
#                  1waterfall
shapviz::sv_waterfall(shapley, row_id = 1)

#                  SHAP
shapresult$shapplotd_facet
shapresult$shapplotd_one

#                  SHAP
shapresult$shapplotc_facet
shapresult$shapplotc_one
shapresult$shapplotc_one2
```

Listing 1: R Script for Model Comparison and Parallel Computation

3 Conclusion

This document provides an R script suitable for comparing multiple machine learning models with parallel computation. The code is ready to be used for model evaluation and preprocessing tasks.
