# Supplementary material for: A prognostic model for highly aggressive prostate cancer using interpretable machine learning techniques
Source: Front Med (Lausanne). 2025 May 12;12:1512870. doi: 10.3389/fmed.2025.1512870 (PMC12104253; doi:10.3389/fmed.2025.1512870)
Supplement: Supplementary file 5 [file Data_Sheet_5.DOCX]

**supplementary materials**

**article title: A Prognostic Model for Highly Aggressive Prostate Cancer Using Interpretable Machine Learning Techniques**

**
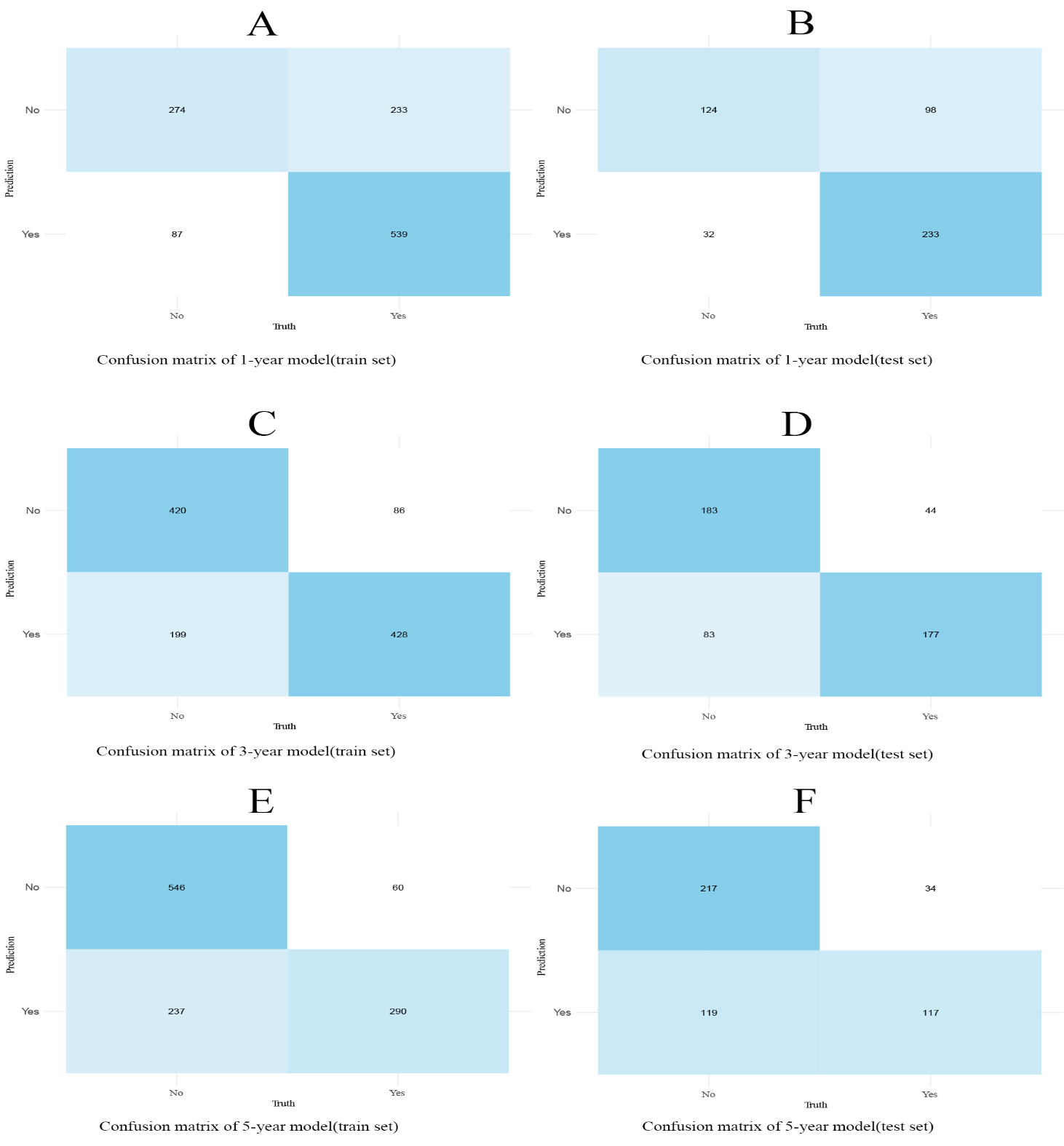
**

**Supplementary Fig.1** Confusion matrix of LightGBM model's predictions in the train and test sets. A.1-year train set; B.1-year test set; C.3-year train set; D.3-year test set; E.5-year train set; F.5-year test set; The matrix shows the counts of true positive (TP), true negative (TN), false positive (FP), and false negative (FN) predictions, with the rows representing actual labels and the columns representing predicted labels. The diagonal elements indicate correct predictions, while the off-diagonal elements show misclassifications. The color intensity reflects the number of instances in each category, providing insight into the model's performance.

**
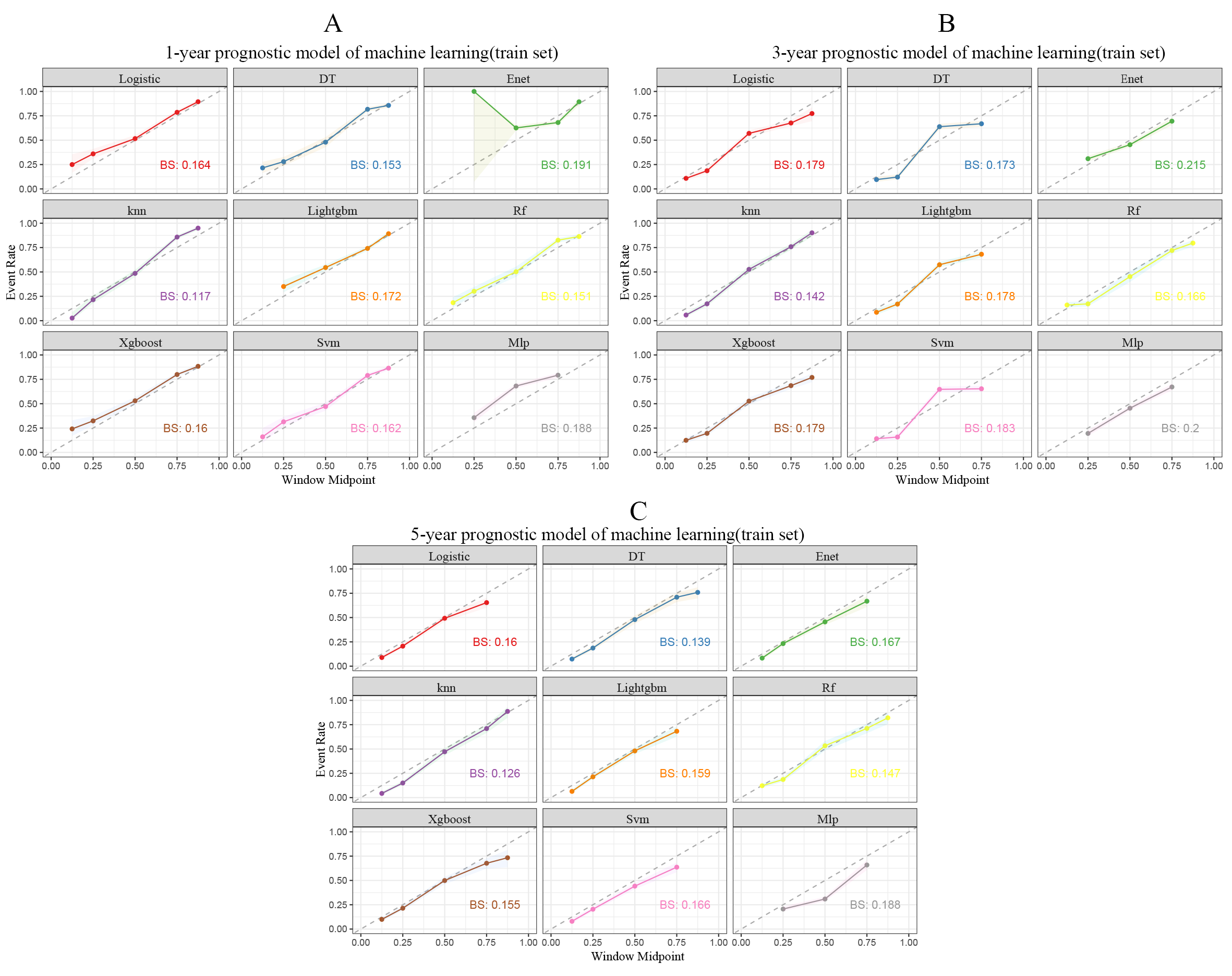
Supplementary Fig.2** Calibration curves based on bier scores for 9 machine learning models in the train sets. A. 1-year prognostic models, B. 3-year prognostic models, C. 5-year prognostic models. The plot illustrates the relationship between the predicted probabilities and the observed outcomes. The x-axis represents the predicted probability, while the y-axis indicates the actual proportion of positive outcomes. A perfectly calibrated model would have a curve that aligns closely with the 45-degree diagonal line, indicating that the predicted probabilities correspond well to the observed frequencies. The Brier score, a measure of model accuracy, is calculated as the mean squared difference between predicted probabilities and actual outcomes. A lower Brier score indicates better model performance, reflecting higher accuracy and calibration. Conversely, a higher Brier score suggests poorer performance, with greater deviation between predictions and observed results.


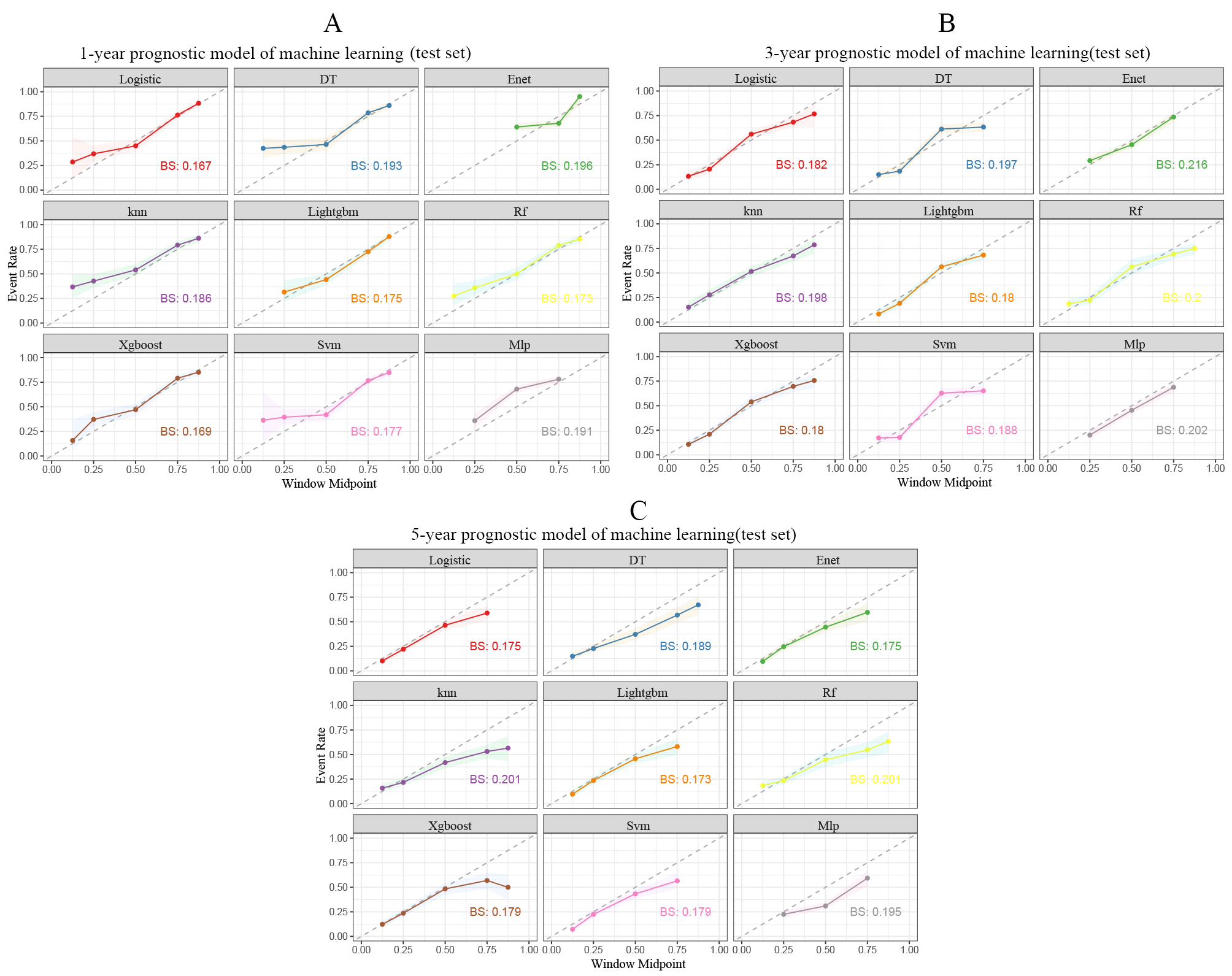


**Supplementary Fig.3** Calibration curves based on bier scores for 9 machine learning models in the test sets. A. 1-year prognostic models, B. 3-year prognostic models, C. 5-year prognostic models. The plot illustrates the relationship between the predicted probabilities and the observed outcomes. The x-axis represents the predicted probability, while the y-axis indicates the actual proportion of positive outcomes. A perfectly calibrated model would have a curve that aligns closely with the 45-degree diagonal line, indicating that the predicted probabilities correspond well to the observed frequencies. The Brier score, a measure of model accuracy, is calculated as the mean squared difference between predicted probabilities and actual outcomes. A lower Brier score indicates better model performance, reflecting higher accuracy and calibration. Conversely, a higher Brier score suggests poorer performance, with greater deviation between predictions and observed results.

**Supplementary Table 1** Performance (F1 Score) of prognostic models built by 9 machine learning algorithms in the test set

|  | 1-year survival | 3-year survival | 5-year survival |  |
| --- | --- | --- | --- | --- |
| **Test set** |  |  |  |  |
| LightGBM | 0.809 | 0.751 | 0.611 |  |
| Logistic | 0.807 | 0.734 | 0.574 |  |
| DT | 0.790 | 0.721 | 0.576 |  |
| Enet | 0.796 | 0.717 | 0.592 |  |
| Knn | 0.765 | 0.646 | 0.540 |  |
| RF | 0.719 | 0.698 | 0.575 |  |
| XGBoost | 0.802 | 0.739 | 0.607 |  |
| SVM | 0.757 | 0.734 | 0.569 |  |
| MLP | 0.780 | 0.736 | 0.575 |  |
| DT: decision tree; ENET:Elastic Net ;KNN:K-Nearest Neighbors; LightGBM: Light Gradient Boosting Machine; RF: Random Forest; XGBoost: Extreme Gradient Boosting; SVM: Support Vector Machine; MLP: Multi-Layer Perceptron. | | | |  |
|  |  |  |  |  |
|  |  |  |  |  |
