# Supplementary material for: A prognostic model for highly aggressive prostate cancer using interpretable machine learning techniques
Source: Front Med (Lausanne). 2025 May 12;12:1512870. doi: 10.3389/fmed.2025.1512870 (PMC12104253; doi:10.3389/fmed.2025.1512870)
Supplement: Supplementary file 6 [file Data_Sheet_6.DOCX]

lightGBM

tree_depth=1, trees=458, learn_rate=0.0059, mtry=5, min_n=10, loss_reduction=0.29

DT

cost_complexity= 0.000639, tree_depth=7, min_n=10

RF

mtry=10, trees=359, min_n=50

Xgboost

tree_depth=1, learn_rate=1.11, mtry=6, min_n=20, loss_reduction=0.869

Enet

penalty=1, mixture=0

MLP

hidden_units=15,penalty=1,epochs=50

SVM

Cost=0.599, rbf_sigma= 0.0174

KNN

Neighbors=11, weight_func=gaussian
